# Supplementary material for: Evaluating the impacts of tiered restrictions introduced in England, during October and December 2020 on COVID-19 cases: a synthetic control study
Source: BMJ Open. 2022 Apr 11;12(4):e054101. doi: 10.1136/bmjopen-2021-054101 (PMC9006191; doi:10.1136/bmjopen-2021-054101)
Supplement: Supplementary data [file bmjopen-2021-054101supp001.pdf]

Appendix 1. Comparison of case numbers at both MSOA and LA levels before and after imputation.

Table A. Summary statistics of the number of cases at both MSOA and LA levels before and after imputation.

		Minimum	1st Quartile	Median	Mean	3rd Quartile	Maximum	Standard deviation	Missing	Number of observations
**October**										
MSOA	Before	3	9	17	21.73	28	1063	24.31	2141	28324
	After	0	8	16	20.29	27	1063	24.02	0	30465
LA	Before	2	269	508	868.5	1049	4291	920.60	0	1089
	After	2	269	508	868.5	1049	4291	920.60	0	1089
**December**										
MSOA	Before	3	10	18	26.68	33	340	25.31	1679	58009
	After	0	10	18	25.97	32	340	25.30	0	59688
LA	Before	15	244.8	507	800.9	946.2	8566	910.36	0	59688
	After	15	244.8	507	800.9	946.2	8566	910.36	0	59688

Appendix 2. Calculating Local Authority level case detection rates.

We use a method proposed by Kulu and Dorey to calculate local authority specific infection rates from data on observed and expected deaths and hospitalisations from Covid-19.[10] The true infection rate was estimated from Covid-19 for each local authority as the number of observed deaths divided by the sum of the expected deaths for each age and sex sub-group, if everyone was infected. The expected deaths for each local authority was calculated by multiplying the population estimates for each age and sex group by the infection fatality rates for that sub-group. The same process was applied using hospitalisations instead of deaths. We use the Infection Fatality and Infection Hospitalisation Rates estimated by Knock et al.[28]

In order to account for varying rates of morbidity between local authorities, following Kulu and Dorey[10] we apply a multiplier of chronic disease prevalence to the expected deaths and hospitalisation for each local authority. The idea being that in areas with higher rates of chronic disease, there would be expected to be higher COVID-19 Infection Fatality and Infection Hospitalisation Rates. Chronic disease was measured as the proportion of the population that had at least one admission to hospital with a diagnosis of cardiovascular disease, chronic respiratory disease, diabetes or chronic kidney disease recorded in their hospital record, which we found in previous research to be highly predictive of COVID-19 mortality.[6] To calculate this multiplier we estimated the increased risk of COVID-19 mortality and hospitalisation associated with chronic illness for each local authority compared to the average using Poisson regression models.

The method outlined above was applied to 7 day moving averages of deaths and hospitalisation for each local authority. As hospital admissions and deaths occur sometime after initial infection, in order to estimate the true infection rate at a specific point in time we need to know the lag between initial case identification and both hospitalisation and death. To estimate this, we ran a set of regression models with observed hospital admissions or deaths as the dependent variable and with observed cases with a range of lags as the predictor variable. We tested lags of 1 to 20 days for hospitalisations and 7 to 30 days for deaths with each different lag tested in a separate model. We determined the best fit lag by selecting the model with the lowest Akaike Information Criterion (AIC). This was 8 days for hospitalisations and 15 days for deaths.

We then divided the 7 day's rolling average of observed cases for each day by the estimated number infected calculated separately using hospitalisation data and deaths data and then took the average

Zhang X, *et al. BMJ Open* 2022; 12:e054101. doi: 10.1136/bmjopen-2021-054101

of the output of the two methods as our measure of case detection rate. Where there was missing data for either hospitalisations or deaths we took the estimated number infected using the method where there was complete data.

Appendix 3. Differences in Tiered restrictions.
Tiered restrictions were introduced at two points in time. Firstly in October LAs in England, were placed in one of three Tiers with restrictions of increasing stringency.26 Tier 1 had the fewest restrictions, groups of up to 6 people were allowed to meet indoors or outdoors. In Tier 2, people were prohibited from mixing inside with individuals outside of their households, were only allowed to meet with up to 6 people outdoors, and pubs and restaurants had to close between 10pm and 5am. In Tier 3, people were additionally prohibited from meeting with people outside their household in private gardens. Pubs and restaurants were only allowed to remain open if they were acting as restaurants and serving a 'substantial meal'. The Tier to which an area was allocated to was based on the average rate of change in case numbers and pressure on the health service across the LA.27 Although explicit criteria have not been published and there is some evidence of pressure from local politicians influencing decisions.28 Following this initial tiered system the whole of England was moved into a month-long national lock down from the 5th November before a return to a three-tier system at the beginning of December.29 This new tiered system had some differences in the restrictions for residents in each tier. The main difference was that in this second period in Tier 2 areas pubs and restaurants were only allowed to stay open if they were serving 'substantial meals', whilst in Tier 3 areas pubs and restaurants had to close except for providing takeaway food. The areas in each tier remained the same until 19th of December, when some areas were re-assigned to a new 'Tier 4' on that day because of rapidly rising case numbers in certain areas, thought to largely be due to a new more infectious variant of the virus, B.1.1.7.11 In Tier 4 areas the guidance was to stay at home except for essential journeys. In both periods and in both Tier 2 and Tier 3, no mixing between households was permitted indoors.

Appendix 4. Replicating analysis only using confirmed cases as an outcome.
We found larger effects when using confirmed COVID-19 cases instead of wider case-detection rates as our outcome in both time periods.

Table B. The comparison of the number of confirmed cases between the Tier 3 and synthetic control Tier 2 areas during the two intervention periods, with lower (LCL) and upper (UCL) 95% confidence limits.

	Percentage change in cases	LCL	UCL	p-value
**October -** All Tier 3	-21%	-28%	-16%	<0.001
**December -** All Tier 3	-28%	-31%	-25%	<0.001

Appendix 5. Sensitivity tests of the spatial spill-over effect.
A concern with localised restrictions in the control of COVID-19 is that there may be spill-over effects, whereby, people move from areas of high restrictions to take advantage of lower restrictions (e.g. visiting restaurants and pubs) in neighbouring areas, increasing transmission in those areas and reducing the overall effectiveness of a system of differential local restrictions. In order to test the spatial spill-over effect between the treatment (Tier 3) and comparison (Tier 2) group, we exclude the Tier 2 MSOA areas locating within 20 km of the treatment group. The distance between MSOA areas is measured by the Euclidean distance of the population weighted centroids of MSOA areas (Data source is ONS Geography Open Data https://geoportal.statistics.gov.uk/datasets/b0a6d8a3dc5d4718b3fd62c548d60f81_0).

Zhang X, *et al. BMJ Open* 2022; 12:e054101. doi: 10.1136/bmjopen-2021-054101

When excluding the Tier 2 MSOA areas located within 20 km of Tier 3 areas we found smaller effects but with high p values (see Table B). This suggests that there may have been some spill-over effects, whereby travel from Tier 3 areas to neighbouring Tier 2 areas contributed to a rise in transmission in neighbouring Tier 2 areas. However, such effects may well have occurred by chance.

Table C. The comparison of the number of cases between the Tier 3 and synthetic control Tier 2 areas during the two intervention periods, excluding the Tier 2 MSOA areas locating within 20 km of the treatment group.

	Percentage change in cases	LCL	UCL	p-value
**October -** All Tier 3	-12%	-25%	3%	0.112
**December -** All Tier 3	-12%	-28%	-2%	0.128

Figure A. Location of areas that entered Tier 3 (yellow) and Tier 2 (purple) at the two intervention time points, excluding the Tier 2 MSOA areas locating within 20 km of the treatment group.

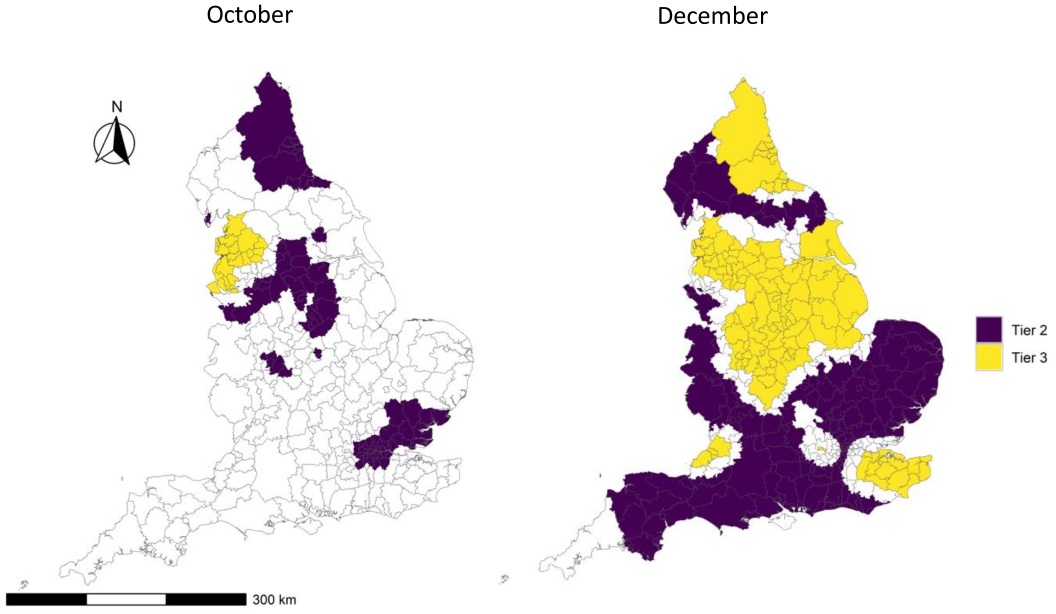

Appendix 6. Weighting of Tier 2 areas used to construct synthetic control group.

Figure B. Weighting of areas that entered Tier 3 (red) and Tier 2 (other colours) to construct synthetic control group at the two intervention time points.

Zhang X, *et al. BMJ Open* 2022; 12:e054101. doi: 10.1136/bmjopen-2021-054101

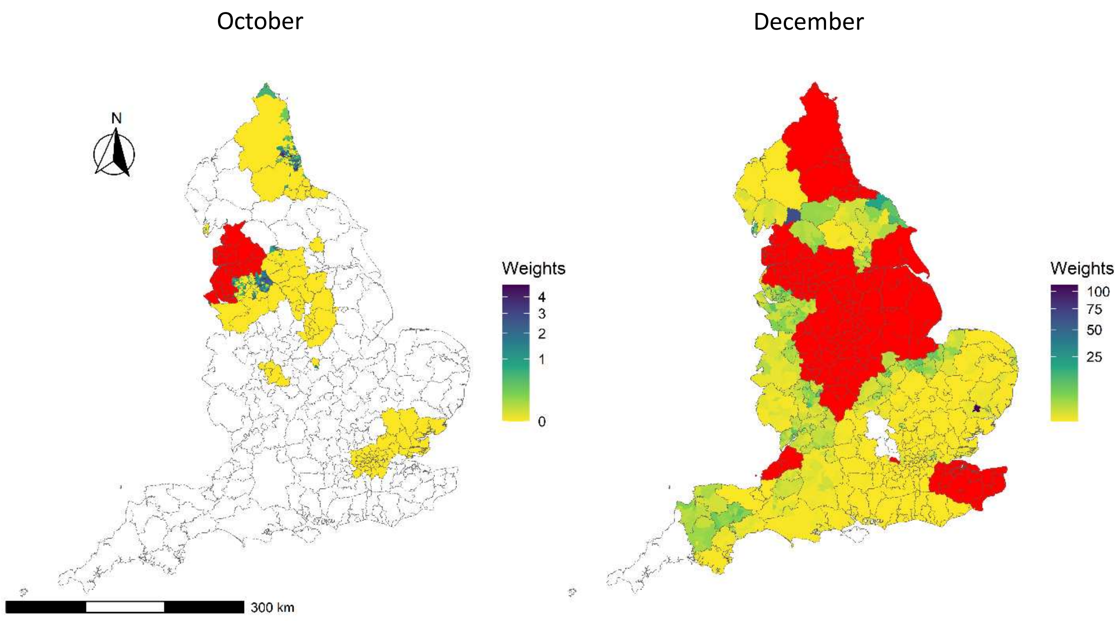

Appendix 7. Placebo permutations.

Comparison of Tier 3 cases following introduction of tiered restrictions in October compared to a synthetic control.

Figure C. October all areas.

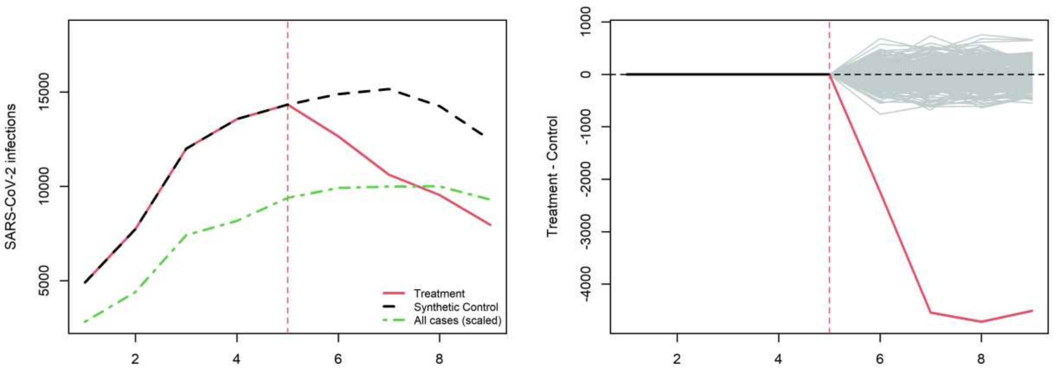

Comparison of Tier 3 cases following introduction of tiered restrictions December compared to a synthetic control.

Figure D. December all areas.

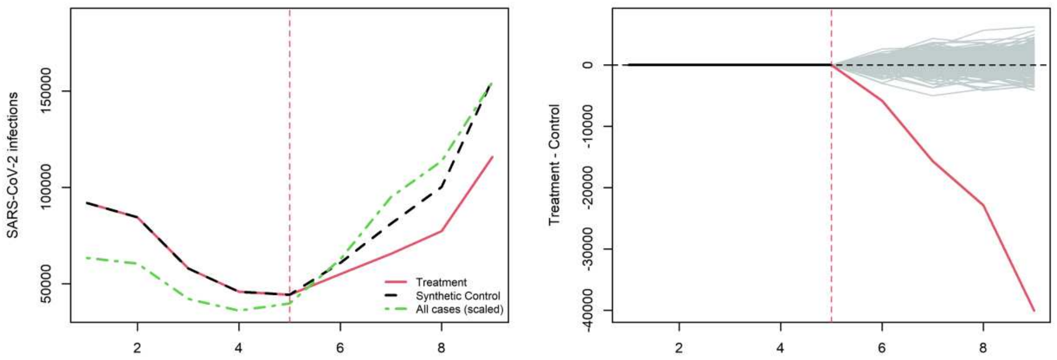
SARS-CoV-2 infections
50000
100000
150000
2
4
6
8
Treatment
Synthetic Control
All cases (scaled)
Treatment - Control
0
-10000
-20000
-30000
-40000

5
