# Supplementary material for: Evaluating the impacts of tiered restrictions introduced in England, during October and December 2020 on COVID-19 cases: a synthetic control study
Source: BMJ Open. 2022 Apr 11;12(4):e054101. doi: 10.1136/bmjopen-2021-054101 (PMC9006191; doi:10.1136/bmjopen-2021-054101)
Supplement: Author's manuscript [file bmjopen-2021-054101.draft_revisions.pdf]

BMJ Open is committed to open peer review. As part of this commitment we make the peer review history of every article we publish publicly available.

When an article is published we post the peer reviewers' comments and the authors' responses online. We also post the versions of the paper that were used during peer review. These are the versions that the peer review comments apply to.

The versions of the paper that follow are the versions that were submitted during the peer review process. They are not the versions of record or the final published versions. They should not be cited or distributed as the published version of this manuscript.

BMJ Open is an open access journal and the full, final, typeset and author-corrected version of record of the manuscript is available on our site with no access controls, subscription charges or pay-per-view fees (http://bmjopen.bmj.com).

If you have any questions on BMJ Open's open peer review process please email info.bmjopen@bmj.com

BMJ Open

Evaluating the impacts of tiered restrictions introduced in England, during October and December 2020 on COVID-19 cases: A synthetic control study

Journal:	*BMJ Open*
Manuscript ID	bmjopen-2021-054101
Article Type:	Original research
Date Submitted by the Author:	04-Jun-2021
Complete List of Authors:	Zhang, Xingna; University of Liverpool Faculty of Health and Life Sciences, Department of Public Health and Policy<br>Owen, Gwilym; University of Liverpool, Department of Public Health and Policy<br>Green, Mark; University of Liverpool, Geography & Planning<br>Buchan, Iain; University of Liverpool, Department of Public Health, Policy and Systems<br>Barr, Ben ; University of Liverpool, Department of Public Health, Policy and Systems
Keywords:	COVID-19, Health policy < HEALTH SERVICES ADMINISTRATION & MANAGEMENT, PUBLIC HEALTH

SCHOLARONE™
Manuscripts

I, the Submitting Author has the right to grant and does grant on behalf of all authors of the Work (as defined in the below author licence), an exclusive licence and/or a non-exclusive licence for contributions from authors who are: i) UK Crown employees; ii) where BMJ has agreed a CC-BY licence shall apply, and/or iii) in accordance with the terms applicable for US Federal Government officers or employees acting as part of their official duties; on a worldwide, perpetual, irrevocable, royalty-free basis to BMJ Publishing Group Ltd ("BMJ") its licensees and where the relevant Journal is co-owned by BMJ to the co-owners of the Journal, to publish the Work in this journal and any other BMJ products and to exploit all rights, as set out in our licence.

The Submitting Author accepts and understands that any supply made under these terms is made by BMJ to the Submitting Author unless you are acting as an employee on behalf of your employer or a postgraduate student of an affiliated institution which is paying any applicable article publishing charge ("APC") for Open Access articles. Where the Submitting Author wishes to make the Work available on an Open Access basis (and intends to pay the relevant APC), the terms of reuse of such Open Access shall be governed by a Creative Commons licence – details of these licences and which Creative Commons licence will apply to this Work are set out in our licence referred to above.

Other than as permitted in any relevant BMJ Author's Self Archiving Policies, I confirm this Work has not been accepted for publication elsewhere, is not being considered for publication elsewhere and does not duplicate material already published. I confirm all authors consent to publication of this Work and authorise the granting of this licence.

For peer review only

Evaluating the impacts of tiered restrictions introduced in England, during October and December 2020 on COVID-19 cases: A synthetic control study

*Xingna Zhang, Gwilym Owen, Mark Green, Iain Buchan, Ben Barr
Public Health, Policy and Systems, Institute of Population Health, University of Liverpool;
Public Health, Policy and Systems, Institute of Population Health, University of Liverpool;
Department of Geography & Planning, University of Liverpool;
Public Health, Policy and Systems, Institute of Population Health, University of Liverpool;
Public Health, Policy and Systems, Institute of Population Health, University of Liverpool

*xingna.zhang@liverpool.ac.uk
Whelan Building, The Quadrangle,
Liverpool L69 3GB,
United Kingdom

Word count (excluding title page, abstract, references, figures and tables): 3,252.

For peer review only - http://bmjopen.bmj.com/site/about/guidelines.xhtml

ABSTRACT

OBJECTIVES
To analyse the impact on SARS-CoV-2 transmission of Tier 3 restrictions introduced in October and December 2020 in England, compared to Tier 2 restrictions, where the main differences were additional restrictions on meeting people outdoors and restrictions on the hospitality sector in Tier 3 areas. We further investigate whether these effects varied between small areas by level of deprivation.

DESIGN
Synthetic control analysis.

SETTING
We identified areas that entered Tier 3 restrictions in October and December, constructed a synthetic control group of places under Tier 2 restrictions, and compared changes in weekly infections over a 4-week period. We used interaction analysis to estimate whether this effect varied by level of deprivation and the prevalence of a new variant (B.1.1.7).

INTERVENTIONS
In both the October and December introductions of tiered restrictions, no mixing between households was permitted indoors in either Tier 2 or Tier 3. The main differences in October was that in Tier 3 people were prohibited from meeting with people outside their household in private gardens and Pubs and restaurants were only allowed to remain open if they were serving a ‘substantial meal’, whilst in Tier 2 people were allowed to meet with up to 6 people in private gardens and all pubs and restaurants remained open. In December, in Tier 3 pubs and restaurants were closed, whilst on Tier 2 only those serving food remained open. The differences in restrictions between Tier 2 and 3 on meeting outside remained the same as in October.

MAIN OUTCOME MEASURE
Weekly reported cases adjusted for changing case-detection rates for neighbourhoods in England.

RESULTS
The introduction of Tier 3 restrictions in October and December was associated with a 14% (95% CI 10% to 19%) and 20% (95% CI 13% to 29%) reduction in infections respectively, compared to the rates expected with Tier 2 restrictions only. The effects were similar across levels of deprivation and by the prevalence of the new variant.

CONCLUSIONS
Compared to Tier 2 restrictions, additional restrictions on hospitality and meeting outdoors introduced in Tier 3 areas in England had a moderate effect on transmission and these restrictions did not appear to increase inequalities in COVID-19 cases.

Article summary
Strengths and limitations of this study

- Our study presents timely empirical evidence of the effectiveness of implementing regional tiered restrictions to manage COVID-19 responses.
- Our paper is the first study to empirically estimate the impact on SARS-CoV-2 infections of the additional restrictions applied to Tier 3 areas in England compared with the counterfactual of having only applied Tier 2 restrictions. We believe our study is also the first to investigate whether such an impact differs by socioeconomic deprivation.

For peer review only - http://bmjopen.bmj.com/site/about/guidelines.xhtml

For peer review only

- We cannot tell from our analysis whether either additional restrictions in Tier 3 on outdoor meeting or the hospitality sector, or a combination of both were responsible for the effects we observed.
- We were only able to adjust for variation in the case detection rate using a relatively crude measure estimated at the LA (Local Authority) level, by assuming constant infection hospitalisation rates and infection fatality rates between places with similar prevalence of underlying health conditions over the study periods.
- We were only able to use data on small neighbourhood areas, rather than on individuals and therefore were unable to investigate how effects of control measures varied by individual or household characteristics – e.g. ethnicity, occupation or household size.

For peer review only

For peer review only - http://bmjopen.bmj.com/site/about/guidelines.xhtml

INTRODUCTION

In Autumn 2020, England experienced a second wave of COVID-19 cases with prevalence increasing 10-fold from 0.1% in August to 1% in October.[1] Reported cases of COVID-19 were unevenly distributed across the country, with areas in the North of England most severely affected. Initially a variety of local restrictions were introduced, which became a standardised three Tier system in October. This was followed by a month-long national lockdown, with tiered restrictions re-introduced in December and a further national lock-down in January 2021.

During the pandemic, evidence has accumulated showing that restrictions such as closing schools, public event bans, and stay-at-home orders, have substantially reduced transmission of SARS-CoV-2.[2] Most evidence is, however, based on national implementation of policies[2,3] and cross-country comparisons,[2] that may not be applicable to restrictions varying across small areas within countries. Much evidence is also based on simulation rather than empirical studies, whereby observed changes in mobility and survey-based indicators of social contact following interventions are used to predict the expected impact of restrictions on cases and hospitalisations using compartmental models.[4] Estimates of intervention impact from such studies may differ from the observed impact on cases, if actual relationships between mobility and transmission following intervention differ from simulation assumptions.

There are also concerns that such restrictions may have differential effects between different socioeconomic contexts. This is because people of disadvantaged communities may not, for example, be able to comply with requirements to work from home due to their occupation or communications of the need to comply with restrictions.[5] Understanding the potential differences in effect of control measures by socioeconomic group is important because the pandemic has disproportionally affected more disadvantaged groups.[6] Interventions designed to control the pandemic may further exacerbate these inequalities if we do not evaluate their differential effects and take actions to mitigate any intervention-generated inequalities.[7]

In this study we analyse the impact on SARS-CoV-2 transmission of Tier 3 restrictions introduced in October and December in England, compared to Tier 2 restrictions, where the main differences were additional restrictions on meeting people outdoors and the hospitality sector in Tier 3 areas. We further investigate whether these effects varied between small areas by level of deprivation.

METHODS

Patient and Public Involvement

Patients and public were not involved.

Data and setting

We use UK government data on weekly number of people with at least one positive COVID-19 test result[8] living in 7201 Middle Layer Super Output Areas (MSOAs), between September 2020 and January 2021. MSOAs are standard geographical units used to report statistics in England, with an average population of 8,000. Where there were fewer than 3 cases in any given week the number of cases was supressed. In these situations, we imputed the number of cases, using complete data available at a higher geographical level (Local Authority), so that the sum of cases across MSOAs within a Local Authority (LA) was equal to the total number of weekly cases reported for that LA. In total, 4% of the outcome data was imputed. LAs are municipalities covering the whole of England and have

For peer review only - http://bmjopen.bmj.com/site/about/guidelines.xhtml

largely been the sub-national geographical units used to organise response, testing and control measures during the pandemic.

As trends in reported cases are affected by changes in testing capacity, testing strategy and public behaviour. We adjusted the weekly cases reported for each MSOA by dividing it by a weekly case detection rate estimate in each LA, calculated using a method outlined by Kulu and Dorey (see Appendix 1).[9]

We also measured local area characteristics that could potentially influence transmission and/or effectiveness of control measures. These included the English Indices of Multiple Deprivation (IMD) - a composite measure of socioeconomic disadvantage,[10] average number of care home beds per capita from the Care Quality Commission, population density, the percentage of the over 70 and 7-11 population using 2019's mid-year population estimates from the Office for National Statistics, the proportion of the Black Asian and Minority Ethnic (BAME) groups and the proportion of students from the 2011 Census. To additionally account for potential differences in testing between areas, we used data for LAs on the number of tests per capita in the 4 weeks prior to the intervention available from the UK government COVID-19 dashboard. To account for differences in the prevalence of the new variant B.1.1.7,[11] we included the proportion of positive tests with S-gene failure on PCR testing for each LA from Public Health England.[12]

This time series of MSOA weekly data, area characteristics and linked LA data, was then merged with a dataset indicating the restrictions that each area was under in any given week. Data on restrictions was compiled and made available from the Open Data Intsitute.[13]

Intervention

In tiered restrictions introduced in both October and December, no mixing between households was permitted indoors in either Tier 2 or Tier 3. The main difference in October was that in Tier 3 people were prohibited from meeting with people outside their household in private gardens and Pubs and restaurants were only allowed to remain open if they were serving a 'substantial meal', whilst in Tier 2 people were allowed to meet with up to 6 people in private gardens and all pubs and restaurants remained open. In December, in Tier 3 pubs and restaurants were closed, whilst in Tier 2 only those serving food remained open. The differences in restrictions between Tier 2 and 3 on meeting outside remained the same as in October. See Appendix 2 for further details.

Analysis

We investigate two intervention time points, weeks commencing 19th October and 7th December. In each period the initial allocation to tiers was announced on Friday and we take the week starting the following Monday as the start of the intervention. We define MSOAs as being in the intervention group if they were in Tier 3 at those time points. We investigate the change in cases in the intervention group, four weeks before and after that time point, compared to a synthetic control group derived from places that entered Tier 2 at the same time. We analyse the two groups based on their initial allocation, even though in October 22% of the MSOAs initially allocated to Tier 2 were later moved to Tier 3. All the MSOAs, apart from those in one local authority (Kent), initially allocated to Tier 3 in December stayed in Tier 3 until the country entered a national lockdown beginning at January 2021. Analysing the groups based on their initial allocation is analogous to an intention to treat analysis in a trial and will provide a more conservative estimate of effect size. This will be less prone to bias of selecting places based on their subsequent transition into Tier 3, which in itself would be influenced by the effectiveness of the tiered restrictions.[14]

We apply the synthetic control method for microdata developed by Robbins et al to estimate the intervention effect.[15,16] The synthetic control method is a generalisation of difference-in-difference

For peer review only - http://bmjopen.bmj.com/site/about/guidelines.xhtml

methods, whereby an untreated version of the treated cases (i.e. a synthetic control) is created using a weighted combination of untreated cases. As the allocation to Tier 3 areas was based on the average level and trend in cases at the LA level this method is able to identify many small areas of Tier 2 that had similar levels and trends in cases as Tier 3 areas before the introduction of restrictions.

To construct the synthetic control group, we derive calibration weights to match the MSOAs in Tier 2 to Tier 3 areas across the four-week period prior to the intervention by local area characteristics described earlier and the corresponding case trends and levels. For the October period we additionally included the number of weeks each area experienced local restrictions before introducing the tiered system. The weighting algorithm derives weights that meet three constraints. Firstly, the sum of weights in the control group equals the number of cases in the intervention group. Secondly, the weighted average of each local area characteristic in the synthetic control group matches those in the intervention group. Lastly, the synthetic control and intervention group also match across all pre-intervention time points in case numbers.[15]

The Average Treatment Effect for the Treated (ATT) is estimated as the difference in cumulative number of cases in the intervention group in the four weeks after the intervention time point, compared to the (weighted) number of cases in the synthetic control group. To estimate the 95% confidence intervals and p-values we apply a permutation procedure, through repeating the analysis through 250 placebo permutations randomly allocating Tier 2 MSOAs to the intervention group.[16] All analysis was performed using R version 4.0.3 and the Microsynth package.[15]

To investigate whether there was a differential effect by socioeconomic group we grouped the MSOAs into 3 equal sized groups (tertiles) by level of deprivation. We then re-ran the weighting algorithm, stratifying the process by IMD tertile. The ATT can be estimated using the calibration weights in a weighted generalised linear model with the binary variable for the intervention group as the exposure. Accounting for the distribution of the outcome data, we fitted a weighted Poisson model with a log link function and the stratified weights, alongside an interaction term between IMD tertile and the intervention indicator.

In December, transmission and potentially the effectiveness of interventions were affected by the emergence of a new variant (B.1.1.7). We used the same approach as above for IMD to investigate differences in the effect of entering Tier 3 in December, based on the prevalence of the new variant in each area.

Sensitivity analysis

To explore sensitivity to different assumptions, we used the confirmed COVID-19 cases instead of wider case-detection rates as our outcome and we found larger effects in both time periods (see Appendix 3). When excluding the Tier 2 MSOA areas located within 20 km of Tier 3 areas we found smaller effects but with high p values (see Appendix 4). This suggests that there may have been some spill-over effects, whereby travel from Tier 3 areas to neighbouring Tier 2 areas contributed to a rise in transmission in neighbouring Tier 2 areas. However, such effects may well have occurred by chance.

RESULTS

Figure 1 shows the areas that entered Tier 3 at the two time points. In October the initial Tier 3 areas (391 MSOAs) were entirely in the North West of England. In December, a larger proportion of England (2,858 MSOAs) initially entered Tier 3.

Table 1. The comparison between the Tier 3 and Tier 2 areas at the two intervention time points in the 4 weeks prior to the introduction of the Tiered system.

For peer review only - http://bmjopen.bmj.com/site/about/guidelines.xhtml

	October		December	
	**Tier 3**	**Tier 2**	**Tier 3**	**Tier 2**
**Average % estimated case detection rate (confirmed cases / estimated number of infections) in 4 weeks before Tiers introduced**	45	52	41	47
**Average tests per 100,000 per week in 4 weeks before Tiers introduced**	3232	2354	2970	2752
**Weekly infections per 100,000 per week in 4 weeks before Tiers introduced**	723	307	784	355
**Care home beds per 10,000 population**	98	69	86	80
**IMD score**	31	25	26	19
**Population density – people per hectare**	32	51	30	41
**% of population 70+**	14	12	14	14
**% population 7-11 years old**	6	6	6	6
**% BAME**	7	22	12	15
**% *S-gene* target failure (SGTF) in routine *PCR***	NA	NA	23	41
**% students**	3	4	3	3
**Total population**	3,068,261	25,272,230	23,347,218	31,682,197
**Number of MSOAs**	391	2994	2858	3774

Table 1 presents summary statistics for areas within each Tier for both time points. As would be expected estimated SARS-CoV-2 infection rates were higher in Tier 3 areas prior to the introduction of the tiered system at both points. Tier 3 areas were more deprived on average and had a lower proportion of the population from BAME groups and lower population density. There were no differences in terms of students and care homes. In December the new variant was more prevalent in Tier 2 than Tier 3 areas as measured by the proportion of S-gene target failure (SGTF) in routine PCR. In constructing the synthetic control group, weights were calculated to minimise the difference in each of the variables in Table 1. As an exact match was achieved the weighted average of each of these variables in the control group was identical to the intervention group. A map showing the geographical pattern of these weights is given in Appendix 5.

Figure 2 shows the trend in infection rates in the intervention and synthetic control areas before and after intervention. As an exact match was achieved these trends were identical in the synthetic control and intervention groups in the pre-intervention period. In October the rates were increasing before the tiered system was introduced. They then started to fall, with the drop starting first in the Tier 3 areas. With the second implementation of the tiered system in December infection rates were falling whilst the country was in national lockdown, then increased as the national lockdown came to an end and the tiered system was re-introduced. This increase however was slower in the Tier 3 areas compared to the synthetic control. See Appendix 6 for charts displaying the differences between outcomes in the intervention and synthetic control groups compared to 250 placebo permutations.

For peer review only - http://bmjopen.bmj.com/site/about/guidelines.xhtml

Table 2 presents the estimated effect of Tier 3 restrictions compared to what would have been expected if Tier 2 restrictions had been applied on those areas. In October the introduction of Tier 3 restrictions was estimated to have led to 14% fewer cases (95% CI 10% to 19%), than what would have been the case if Tier 2 restrictions would have been applied. In December, the Tier 3 restrictions are estimated to have led to a slightly greater reduction in cases of 20% (95% CI 13% to 29%).

In the subgroup analysis by deprivation there is a statistically significant effect for each level of deprivation for each time period, suggesting that Tier 3 interventions had an effect across all levels of deprivation. In October the effect estimate tended to be greater the more deprived an area was, although there was a high probability that this difference occurred by chance with high p-values. In December, the effect was similar across all levels of deprivation and no interaction effects were detected again. Including an interaction term between the proportion of cases that were the new variant B.1.1.7 and the intervention group, suggested that the effect of Tier 3 restrictions may have been greater in areas where the new variant was more prevalent (-27%, 95% CIs –35% to –18%), but again the p-values for this interaction were greater than 0.05, indicating substantial uncertainty.

Table 2. Results of the synthetic control analysis – indicating the relative reduction in infections in Tier 3 areas compared to what would have been expected if Tier 2 restrictions had been applied. Interaction analysis shows differences in effect by level of deprivation and prevalence of variant B.1.1.7 indicated by S-gene target failure (SGTF) where quantitative reverse transcriptase PCR is used for COVID-19 diagnosis.

	Percentage change in cases	95% CI		p-value	p-value for interaction in subgroup analysis
		LCL	UCL		
**October** - All Tier 3	-14%	-19%	-10%	<0.001	
Most affluent areas	-10%	-17%	-2%	0.016	
Intermediate deprivation	-15%	-22%	-7%	<0.001	0.354
Most deprived areas	-19%	-29%	-7%	0.003	0.214
**December** - All Tier 3	-20%	-29%	-13%	<0.001	
Most affluent areas	-19%	-24%	-14%	<0.001	
Intermediate deprivation	-26%	-38%	-11%	0.001	0.362
Most deprived areas	-14%	-26%	0%	0.046	0.448
Low SGTF (2%-20%)	-13%	-30%	9%	0.236	
Intermediate SGTF (21%-44%)	-6%	-16%	5%	0.271	0.579
High SGTF (45%-85%)	-27%	-35%	-18%	<0.001	0.174

DISCUSSION

Our study presents timely empirical evidence of the effectiveness of implementing regional tiered restrictions to manage COVID-19 responses. We find more stringent regional restrictions effective at reducing infection rates. For both time periods, areas placed in Tier 3 restrictions experienced a moderate reduction in SARS-CoV-2 infections compared to what would have been expected if the same areas had been placed into Tier 2. This is consistent with reporting from the UK Scientific Advisory Group on Emergencies (SAGE) – which concluded in November that the October Tier 3

For peer review only - http://bmjopen.bmj.com/site/about/guidelines.xhtml

restrictions had reduced transmission, although they concluded that the effect could not be quantified and that it was unclear if Tier 3 restrictions alone would be sufficient to reduce R below 1.[17] One simulation study predicted that moving into Tier 3 restrictions in England in October reduced the effective reproduction number (R) by 10%[4] which is similar to the effect size we estimate here.

This study adds to the evidence that the additional restrictions on outdoor meeting and the hospitality sector have an important role to play in controlling transmission. The two main differences between Tier 3 and Tier 2 were that people in Tier 3 were not allowed to meet with people outside their household in private gardens and there were greater restrictions on pubs and restaurants. We cannot tell from our analysis whether either additional restrictions, or a combination of both were responsible for the effects we observed. There is, however, some previous evidence indicating that that hospitality settings do play a role in transmission,[18–21] and outdoor proximity is thought to have a low risk of transmission.[22] We find no consistent evidence that the effects differed by levels of deprivation. The lack of differential effects may be because the differences of restrictions in this study largely affect the hospitality sector and outdoor gathering, rather than for example employment in other sectors, and involvement in these activities may not differ markedly between socioeconomic groups.

The emergence of new more infectious variants of SARS-CoV-2 raises concerns regarding the effectiveness of existing control measures. Increased infectiousness could mean that some activities such as outdoor contact previously seen as low risk could become higher risk. Our results suggest that it is plausible that in the second period of our study, when the new variant B.1.1.7 was more prevalent, the marginal effect of restrictions on outdoor meeting and hospitality settings was relatively greater. There was however, high statistical uncertainty with this finding – possibly inflated by variant surveillance being more accurate in some areas than in others. Further investigation is needed to understand the effectiveness of these restrictions in the presence of new variants.

Our analysis has some limitations. Firstly, we were only able to adjust for variation in the case detection rate using a relatively crude measure estimated at the LA level. This estimate assumes that the infection hospitalisation rate and the infection fatality rate do not vary between places that have similar prevalence of underlying health conditions and do not vary over the study periods. Our analysis also assumes that the case detection rate is constant across MSOAs within each LA. We do however find larger effects when not applying our estimated case detection rate and we also adjusted for differences in the amount of testing carried out in each area. Secondly, although we were able to match areas to ensure a good balance of potential confounding factors prior to the intervention, it is still possible that unmeasured variables could bias the results. Thirdly, we were only able to use data on small neighbourhood areas, rather than on individuals and therefore were unable to investigate how effects of control measures varied by individual or household characteristics.

As countries such as the UK continue the battle to control COVID-19 cases, with large regional differences in transmission, tiered restrictions as well as national lockdowns will likely be needed to reduce infection levels whilst sufficient effective vaccination coverage is achieved. At present the UK government's plans for exiting the current lock down are for a staged easing of restrictions at the same time across the whole of England.[23] Concerns have been raised about this – one size fits all – approach, given that high infection rates persist – particularly in more deprived areas.[24] Our analysis indicates that tiered restrictions in outdoor gathering and in the hospitality sector are effective at moderately reducing the growth of cases and could be part of an effective strategy for reducing geographical differences in transmission risk as we emerge from the pandemic.

For peer review only - http://bmjopen.bmj.com/site/about/guidelines.xhtml

For peer review only

Author contribution statement

B.B. conceived the original Idea, devised the project, the main conceptual ideas and proof outline. X.Z. and B.B. carried out the data analysis. X.Z. drafted the manuscript and designed the figures with support from G.O. and B.B.. G.O. aided in improving the model specification, and M.G. aided in interpreting the results and commented on the manuscript. I.B. aided in supervising the project and commented on the manuscript. All authors provided critical feedback and helped to shape the research manuscript.

Conflicts of interest statement

We have no conflicts of interest to disclose.

Ethics statements

Patient consent for publication

Not required.

Ethics approval

University Research Ethics Committee review is not required by the University of Liverpool for the secondary analysis of data which have been anonymised by an external party and are provided to the research team in a fully anonymised format.

Data sharing statement

All the data we used are publicly accessible and statistical code is available from the authors upon request.

Acknowledgement

This study is funded by the National Institute for Health Research (NIHR) Health Protection Research Unit in Gastrointestinal Infections, a partnership between Public Health England, the University of Liverpool and the University of Warwick. Grant No: NIHR ref NIHR200910. The views expressed are those of the author(s) and not necessarily those of the NIHR, Public Health England or the Department of Health and Social Care. BB is also supported by the NIHR Applied Research Collaboration North West Coast (ARC NWC). GO is supported by the NIHR School for Public Health Research. IB is supported by NIHR Senior Investigator award. The views expressed in this publication are those of the author(s) and not necessarily those of the NIHR or the Department of Health and Social Care.

For peer review only

For peer review only - http://bmjopen.bmj.com/site/about/guidelines.xhtml

References

1. Office for National Statistics. Coronavirus (COVID-19) Infection Survey, UK: 30 October 2020. 2020. https://www.ons.gov.uk/peoplepopulationandcommunity/healthandsocialcare/conditionsanddiseases/bulletins/coronaviruscovid19infectionsurveypilot/30october2020

2. Li Y, Campbell H, Kulkarni D, Harpur A, Nundy M, Wang X, et al. The temporal association of introducing and lifting non-pharmaceutical interventions with the time-varying reproduction number (R) of SARS-CoV-2: a modelling study across 131 countries. *Lancet Infect Dis*. 2021 Feb 1;21(2):193–202.

3. Davies NG, Kucharski AJ, Eggo RM, Gimma A, Edmunds WJ, Jombart T, et al. Effects of non-pharmaceutical interventions on COVID-19 cases, deaths, and demand for hospital services in the UK: a modelling study. *Lancet Public Health*. 2020 Jul 1;5(7):e375–85.

4. Davies NG, Barnard RC, Jarvis CI, Russell TW, Semple MG, Jit M, et al. Association of tiered restrictions and a second lockdown with COVID-19 deaths and hospital admissions in England: a modelling study. *Lancet Infect Dis*. 2020 Dec 23 (accessed 2021 Jan 6);0(0). https://www.thelancet.com/journals/laninf/article/PIIS1473-3099(20)30984-1/abstract

5. Davidson EM, Liu JJ, Bhopal R, White M, Johnson MR, Netto G, et al. Behavior Change Interventions to Improve the Health of Racial and Ethnic Minority Populations: A Tool Kit of Adaptation Approaches. *Milbank Q*. 2013 Dec;91(4):811–51.

6. Daras K, Alexiou A, Rose TC, Buchan I, Taylor-Robinson D, Barr B. How does vulnerability to COVID-19 vary between communities in England? Developing a Small Area Vulnerability Index (SAVI). *J Epidemiol Community Health*. 2021 Feb 4 (accessed 2021 Feb 26); http://jech.bmj.com/content/early/2021/02/04/jech-2020-215227

7. Lorenc T, Petticrew M, Welch V, Tugwell P. What types of interventions generate inequalities? Evidence from systematic reviews. *J Epidemiol Community Health*. 2013 Feb;67(2):190–3.

8. Official UK Coronavirus Dashboard. (accessed 2021 Jan 28). https://coronavirus.data.gov.uk/details/cases

9. Kulu H, Dorey P. Infection rates from Covid-19 in Great Britain by geographical units: A model-based estimation from mortality data. *Health Place*. 2021 Jan;67:102460.

10. Ministry of Housing C& LG. English indices of deprivation 2019: research report. GOV.UK. 2019. https://www.gov.uk/government/publications/english-indices-of-deprivation-2019-research-report

11. Prime Minister's Office, 10 Downing Street. Prime Minister announces Tier 4: 'Stay At Home' Alert Level in response to new COVID variant. 2020 (accessed 2021 Jan 25). https://www.gov.uk/government/news/prime-minister-announces-tier-4-stay-at-home-alert-level-in-response-to-new-covid-variant

12. Kirby T. New variant of SARS-CoV-2 in UK causes surge of COVID-19. *Lancet Respir Med*. 2021 Jan 5 (accessed 2021 Jan 11);0(0). https://www.thelancet.com/journals/lanres/article/PIIS2213-2600(21)00005-9/abstract

For peer review only - http://bmjopen.bmj.com/site/about/guidelines.xhtml

13. Tennison J. Local coronavirus restrictions in England. Google Docs. (accessed 2021 Jan 28). https://docs.google.com/spreadsheets/d/1HBVmvsQXrkQgySW_OiTQdrS8WGCXqgWnmZ43PPi0XgY/edit?usp=embed_facebook

14. Cole SR, Platt RW, Schisterman EF, Chu H, Westreich D, Richardson D, et al. Illustrating bias due to conditioning on a collider. *Int J Epidemiol*. 2010 accepted;39(2):417–20.

15. Robbins MW, Davenport S. microsynth: Synthetic Control Methods for Disaggregated and Micro-Level Data in R. *J Stat Softw Vol 1 Issue 2 2021*. 2021 Jan 14; https://www.jstatsoft.org/v097/i02

16. Robbins MW, Saunders J, Kilmer B. A Framework for Synthetic Control Methods With High-Dimensional, Micro-Level Data: Evaluating a Neighborhood-Specific Crime Intervention. *J Am Stat Assoc*. 2017 Jan 2;112(517):109–26.

17. SAGE 67 minutes: Coronavirus (COVID-19) response, 12 November 2020. GOV.UK. (accessed 2021 Feb 2). https://www.gov.uk/government/publications/sage-67-minutes-coronavirus-covid-19-response-12-november-2020

18. Thompson H, Mousa A, Dighe A, Fu H, Arnedo-Pena A, Barrett P, et al. Report 38: SARS-CoV-2 setting-specific transmission rates: a systematic review and meta-analysis. 22. 2020 Nov (accessed 2021 Feb 2). http://spiral.imperial.ac.uk/handle/10044/1/84270

19. Koh WC, Naing L, Chaw L, Rosledzana MA, Alikhan MF, Jamaludin SA, et al. What do we know about SARS-CoV-2 transmission? A systematic review and meta-analysis of the secondary attack rate and associated risk factors. *PLOS ONE*. 2020 Oct 8;15(10):e0240205.

20. Fisher KA. Community and Close Contact Exposures Associated with COVID-19 Among Symptomatic Adults ≥18 Years in 11 Outpatient Health Care Facilities — United States, July 2020. *MMWR Morb Mortal Wkly Rep*. 2020 (accessed 2021 Feb 2);69. https://www.cdc.gov/mmwr/volumes/69/wr/mm6936a5.htm

21. Qiu X, Nergiz AI, Maraolo AE, Bogoch II, Low N, Cevik M. Defining the role of asymptomatic and pre-symptomatic SARS-CoV-2 transmission - a living systematic review. *Clin Microbiol Infect Off Publ Eur Soc Clin Microbiol Infect Dis*. 2021 Jan 20;

22. Bulfone TC, Malekinejad M, Rutherford GW, Razani N. Outdoor Transmission of SARS-CoV-2 and Other Respiratory Viruses: A Systematic Review. *J Infect Dis*. (accessed 2021 Feb 2); https://academic.oup.com/jid/advance-article/doi/10.1093/infdis/jiaa742/6009483

23. COVID-19 Response - Spring 2021. GOV.UK. (accessed 2021 Mar 1). https://www.gov.uk/government/publications/covid-19-response-spring-2021

24. Is England's exit plan from lockdown a road to nowhere? The BMJ. 2021 (accessed 2021 Mar 1). https://blogs.bmj.com/bmj/2021/02/25/is-englands-exit-plan-from-lockdown-a-road-to-nowhere/

25. Knock E, Whittles L, Lees J, Perez GP, Verity R, Fitzjohn R, et al. Report 41: The 2020 SARS-CoV-2 epidemic in England: key epidemiological drivers and impact of interventions. 2020. http://dx.doi.org/10.25561/85146

For peer review only - http://bmjopen.bmj.com/site/about/guidelines.xhtml

26. Scott E. Covid-19 local alert levels: Three-tier system for England. 2020 Oct 13 (accessed 2021 Jan 14); https://lordslibrary.parliament.uk/covid-19-local-alert-levels-three-tier-system-for-england/

27. Department of Health and Social Care. Full list of local restriction tiers by area. 2020 (accessed 2021 Jan 26). https://www.gov.uk/guidance/full-list-of-local-restriction-tiers-by-area

28. 'Row over Tier 3 Covid rule not just about Greater Manchester'. ITV News. 2020 (accessed 2021 Jan 28). https://www.itv.com/news/2020-10-18/row-over-tier-3-covid-rule-not-just-about-greater-manchester-says-mayor-andy-burnham

29. Cabinet Office. Covid-19 Winter Plan. 2020 (accessed 2021 Jan 25). https://www.gov.uk/government/publications/covid-19-winter-plan/covid-19-winter-plan

For peer review only

For peer review only - http://bmjopen.bmj.com/site/about/guidelines.xhtml

Figure 1. Location of areas that entered Tier 3 (yellow) and Tier 2 (purple) at the two intervention time points.

Figure 2. The trend in case rates with their 95% confidence intervals in the Tier 3 areas and in the synthetic control group.

For peer review only

For peer review only - http://bmjopen.bmj.com/site/about/guidelines.xhtml

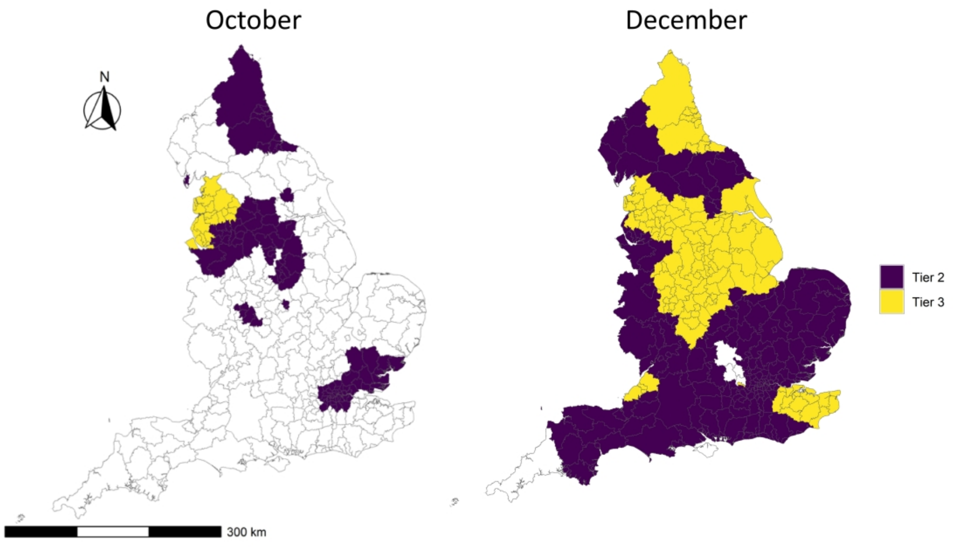

Figure 1. Location of areas that entered Tier 3 (yellow) and Tier 2 (purple) at the two intervention time points.

338x190mm (300 x 300 DPI)

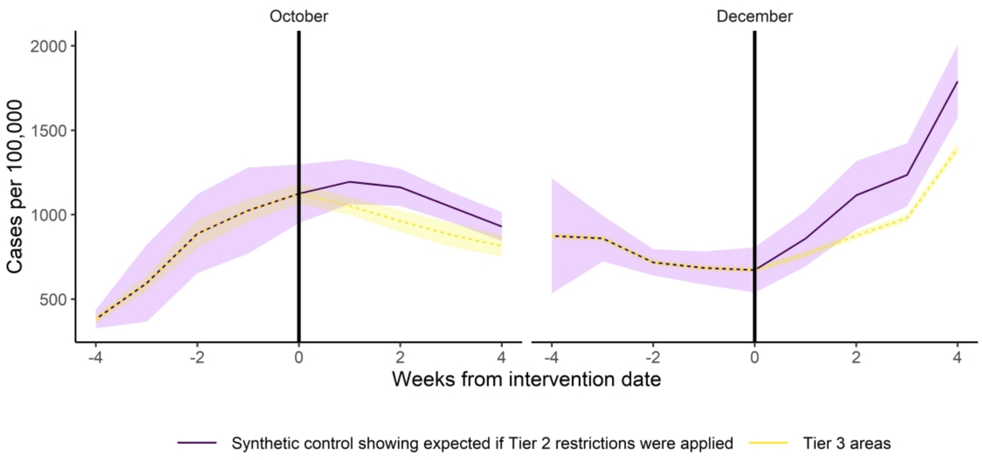

Figure 2. The trend in case rates with their 95% confidence intervals in the Tier 3 areas and in the synthetic control group.

338x190mm (300 x 300 DPI)

Appendices.

Appendix 1. Calculating Local Authority level case detection rates.

We use a method proposed by Kulu and Dorey to calculate local authority specific infection rates from data on observed and expected deaths and hospitalisations from Covid-19.[9] The true infection rate was estimated from Covid-19 for each local authority as the number of observed deaths divided by the sum of the expected deaths for each age and sex sub-group, if everyone was infected. The expected deaths for each local authority was calculated by multiplying the population estimates for each age and sex group by the infection fatality rates for that sub-group. The same process was applied using hospitalisations instead of deaths. We use the Infection Fatality and Infection Hospitalisation Rates estimated by Knock et al.[25]

In order to account for varying rates of morbidity between local authorities, following Kulu and Dorey[9] we apply a multiplier of chronic disease prevalence to the expected deaths and hospitalisation for each local authority. The idea being that in areas with higher rates of chronic disease, there would be expected to be higher COVID-19 Infection Fatality and Infection Hospitalisation Rates. Chronic disease was measured as the proportion of the population that had at least one admission to hospital with a diagnosis of cardiovascular disease, chronic respiratory disease, diabetes or chronic kidney disease recorded in their hospital record, which we found in previous research to be highly predictive of COVID-19 mortality.[6] To calculate this multiplier we estimated the increased risk of COVID-19 mortality and hospitalisation associated with chronic illness for each local authority compared to the average using Poisson regression models.

The method outlined above was applied to 7 day moving averages of deaths and hospitalisation for each local authority. As hospital admissions and deaths occur sometime after initial infection, in order to estimate the true infection rate at a specific point in time we need to know the lag between initial case identification and both hospitalisation and death. To estimate this, we ran a set of regression models with observed hospital admissions or deaths as the dependent variable and with observed cases with a range of lags as the predictor variable. We tested lags of 1 to 20 days for hospitalisations and 7 to 30 days for deaths with each different lag tested in a separate model. We determined the best fit lag by selecting the model with the lowest Akaike Information Criterion (AIC). This was 8 days for hospitalisations and 15 days for deaths.

We then divided the 7 day's rolling average of observed cases for each day by the estimated number infected calculated separately using hospitalisation data and deaths data and then took the average of the output of the two methods as our measure of case detection rate. Where there was missing data for either hospitalisations or deaths we took the estimated number infected using the method where there was complete data.

Appendix 2. Differences in Tiered restrictions.

Tiered restrictions were introduced at two points in time. Firstly in October LAs in England, were placed in one of three Tiers with restrictions of increasing stringency.[26] Tier 1 had the fewest restrictions, groups of up to 6 people were allowed to meet indoors or outdoors. In Tier 2, people were prohibited from mixing inside with individuals outside of their households, were only allowed to meet with up to 6 people outdoors, and pubs and restaurants had to close between 10pm and 5am. In Tier 3, people were additionally prohibited from meeting with people outside their household in private gardens. Pubs and restaurants were only allowed to remain open if they were acting as restaurants and serving a 'substantial meal'. The Tier to which an area was allocated to was based on the average rate of change in case numbers and pressure on the health service across the LA.[27] Although explicit criteria have not been published and there is some evidence of pressure from local politicians influencing decisions.[28] Following this initial tiered system the whole of England was moved

For peer review only - http://bmjopen.bmj.com/site/about/guidelines.xhtml

into a month-long national lock down from the 5th November before a return to a three-tier system at the beginning of December.[29] This new tiered system had some differences in the restrictions for residents in each tier. The main difference was that in this second period in Tier 2 areas pubs and restaurants were only allowed to stay open if they were serving 'substantial meals', whilst in Tier 3 areas pubs and restaurants had to close except for providing takeaway food. The areas in each tier remained the same until 19th of December, when some areas were re-assigned to a new 'Tier 4' on that day because of rapidly rising case numbers in certain areas, thought to largely be due to a new more infectious variant of the virus, B.1.1.7.[11] In Tier 4 areas the guidance was to stay at home except for essential journeys. In both periods and in both Tier 2 and Tier 3, no mixing between households was permitted indoors.

Appendix 3. Replicating analysis only using confirmed cases as an outcome.
We found larger effects when using confirmed COVID-19 cases instead of wider case-detection rates as our outcome in both time periods.

Table A. The comparison of the number of confirmed cases between the Tier 3 and synthetic control Tier 2 areas during the two intervention periods, with lower (LCL) and upper (UCL) 95% confidence limits.

	Percentage change in cases	LCL	UCL	p-value
**October** - All Tier 3	-21%	-28%	-16%	<0.001
**December** - All Tier 3	-28%	-31%	-25%	<0.001

Appendix 4. Sensitivity tests of the spatial spill-over effect.
A concern with localised restrictions in the control of COVID-19 is that there may be spill-over effects, whereby, people move from areas of high restrictions to take advantage of lower restrictions (e.g. visiting restaurants and pubs) in neighbouring areas, increasing transmission in those areas and reducing the overall effectiveness of a system of differential local restrictions. In order to test the spatial spill-over effect between the treatment (Tier 3) and comparison (Tier 2) group, we exclude the Tier 2 MSOA areas locating within 20 km of the treatment group. The distance between MSOA areas is measured by the Euclidean distance of the population weighted centroids of MSOA areas (Data source is ONS Geography Open Data https://geoportal.statistics.gov.uk/datasets/b0a6d8a3dc5d4718b3fd62c548d60f81_0).

When excluding the Tier 2 MSOA areas located within 20 km of Tier 3 areas we found smaller effects but with high p values (see Table B). This suggests that there may have been some spill-over effects, whereby travel from Tier 3 areas to neighbouring Tier 2 areas contributed to a rise in transmission in neighbouring Tier 2 areas. However, such effects may well have occurred by chance.

Table B. The comparison of the number of cases between the Tier 3 and synthetic control Tier 2 areas during the two intervention periods, excluding the Tier 2 MSOA areas locating within 20 km of the treatment group.

	Percentage change in cases	LCL	UCL	p-value
**October** - All Tier 3	-12%	-25%	3%	0.112
**December** - All Tier 3	-12%	-28%	-2%	0.128

For peer review only - http://bmjopen.bmj.com/site/about/guidelines.xhtml

Figure A. Location of areas that entered Tier 3 (yellow) and Tier 2 (purple) at the two intervention time points, excluding the Tier 2 MSOA areas locating within 20 km of the treatment group.

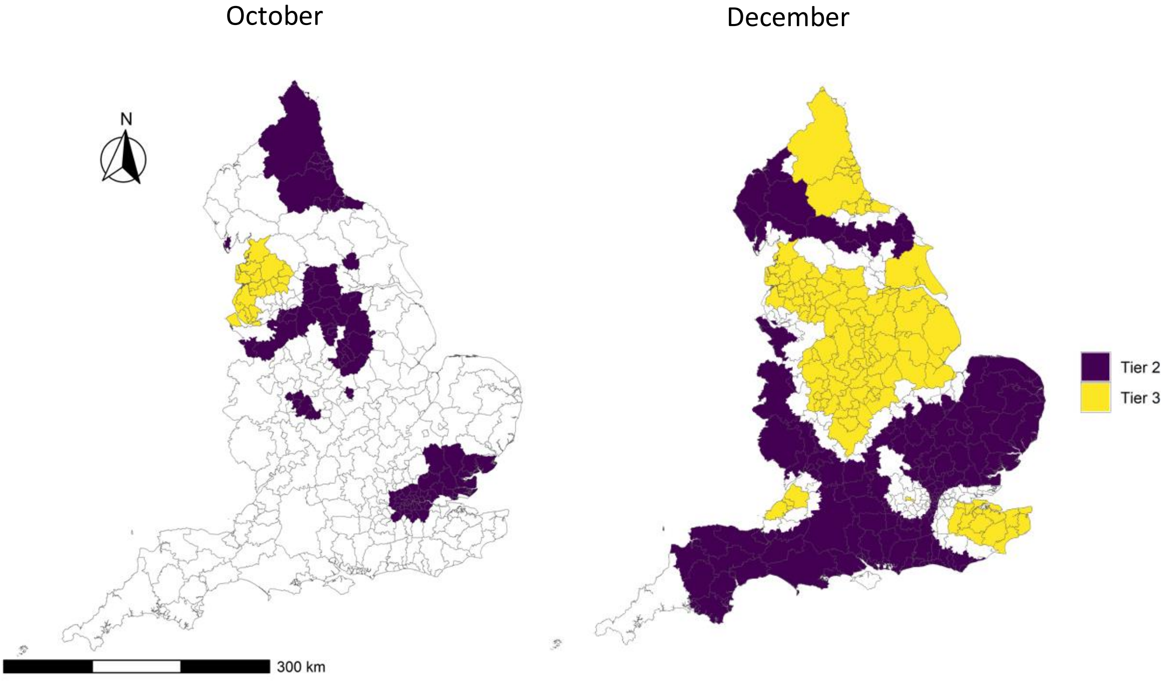

Appendix 5. Weighting of Tier 2 areas used to construct synthetic control group.

Figure B. Weighting of areas that entered Tier 3 (red) and Tier 2 (other colours) to construct synthetic control group at the two intervention time points.

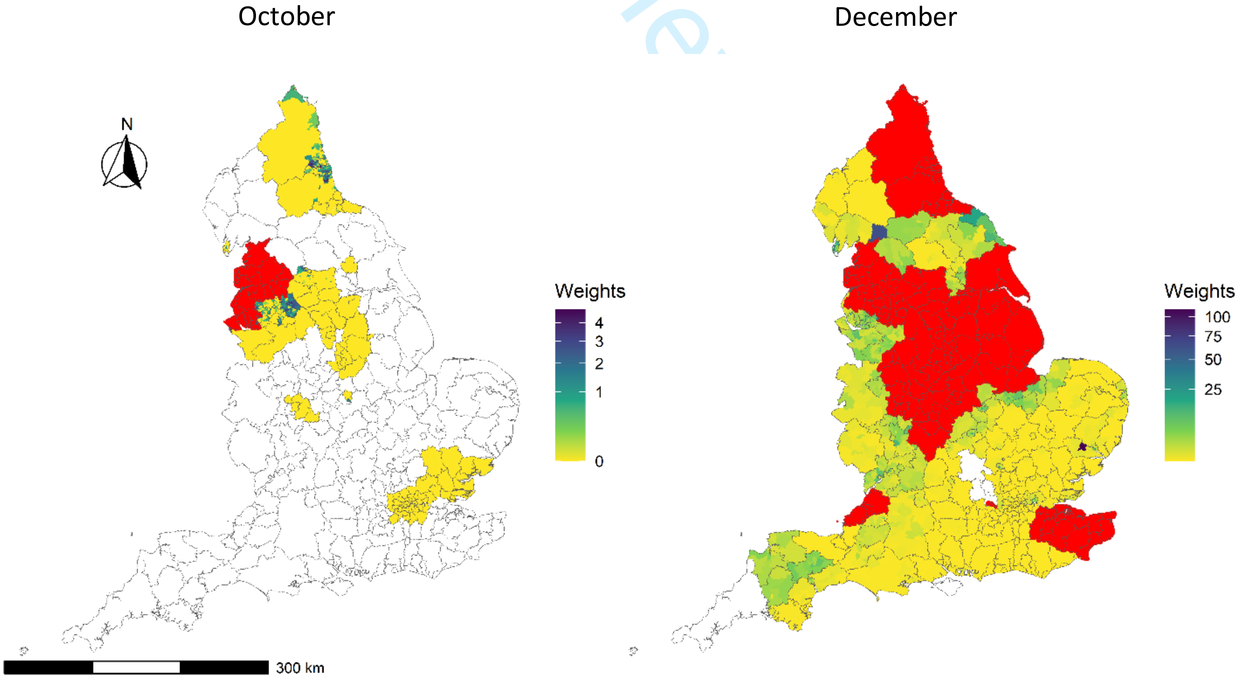

Appendix 6. Placebo permutations.

For peer review only - http://bmjopen.bmj.com/site/about/guidelines.xhtml

Comparison of Tier 3 cases following introduction of tiered restrictions in October compared to a synthetic control.

Figure C. October all areas.

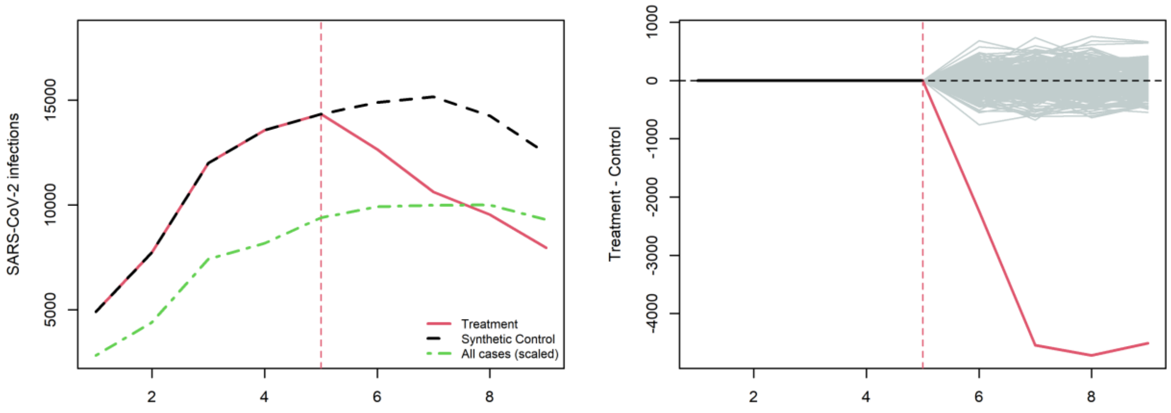

Comparison of Tier 3 cases following introduction of tiered restrictions December compared to a synthetic control.

Figure D. December all areas.

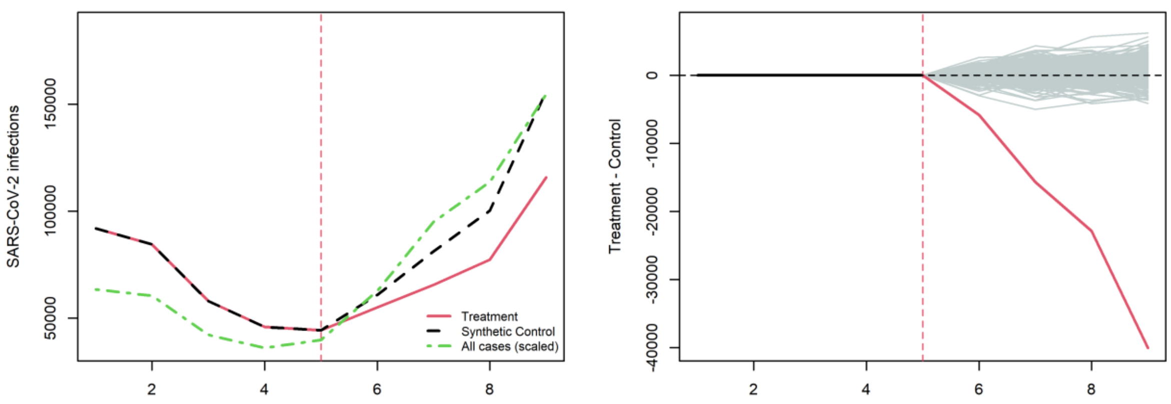

For peer review only - http://bmjopen.bmj.com/site/about/guidelines.xhtml

STROBE 2007 (v4) Statement—Checklist of items that should be included in reports of *cross-sectional studies*

Section/Topic	Item #	Recommendation	Reported on page #
**Title and abstract**	1	(*a*) Indicate the study's design with a commonly used term in the title or the abstract	1
		(*b*) Provide in the abstract an informative and balanced summary of what was done and what was found	2-3
**Introduction**			
Background/rationale	2	Explain the scientific background and rationale for the investigation being reported	4
Objectives	3	State specific objectives, including any prespecified hypotheses	4
**Methods**			
Study design	4	Present key elements of study design early in the paper	4
Setting	5	Describe the setting, locations, and relevant dates, including periods of recruitment, exposure, follow-up, and data collection	4-5
Participants	6	(*a*) Give the eligibility criteria, and the sources and methods of selection of participants	4-6
Variables	7	Clearly define all outcomes, exposures, predictors, potential confounders, and effect modifiers. Give diagnostic criteria, if applicable	5-6
Data sources/ measurement	8*	For each variable of interest, give sources of data and details of methods of assessment (measurement). Describe comparability of assessment methods if there is more than one group	4-5, 7
Bias	9	Describe any efforts to address potential sources of bias	*6*
Study size	10	Explain how the study size was arrived at	7
Quantitative variables	11	Explain how quantitative variables were handled in the analyses. If applicable, describe which groupings were chosen and why	4-8
Statistical methods	12	(*a*) Describe all statistical methods, including those used to control for confounding	5-8
		(*b*) Describe any methods used to examine subgroups and interactions	8
		(*c*) Explain how missing data were addressed	4
		(*d*) If applicable, describe analytical methods taking account of sampling strategy	
		(*e*) Describe any sensitivity analyses	6
**Results**			

For peer review only

Participants	13*	(a) Report numbers of individuals at each stage of study—eg numbers potentially eligible, examined for eligibility, confirmed eligible, included in the study, completing follow-up, and analysed	7
		(b) Give reasons for non-participation at each stage	7
		(c) Consider use of a flow diagram	
Descriptive data	14*	(a) Give characteristics of study participants (eg demographic, clinical, social) and information on exposures and potential confounders	7
		(b) Indicate number of participants with missing data for each variable of interest	
Outcome data	15*	Report numbers of outcome events or summary measures	7-8
Main results	16	(*a*) Give unadjusted estimates and, if applicable, confounder-adjusted estimates and their precision (eg, 95% confidence interval). Make clear which confounders were adjusted for and why they were included	8
		(*b*) Report category boundaries when continuous variables were categorized	8
		(*c*) If relevant, consider translating estimates of relative risk into absolute risk for a meaningful time period	
Other analyses	17	Report other analyses done—eg analyses of subgroups and interactions, and sensitivity analyses	8
**Discussion**			
Key results	18	Summarise key results with reference to study objectives	8-9
Limitations	19	Discuss limitations of the study, taking into account sources of potential bias or imprecision. Discuss both direction and magnitude of any potential bias	9
Interpretation	20	Give a cautious overall interpretation of results considering objectives, limitations, multiplicity of analyses, results from similar studies, and other relevant evidence	8-9
Generalisability	21	Discuss the generalisability (external validity) of the study results	9
**Other information**			
Funding	22	Give the source of funding and the role of the funders for the present study and, if applicable, for the original study on which the present article is based	10

*Give information separately for cases and controls in case-control studies and, if applicable, for exposed and unexposed groups in cohort and cross-sectional studies.

Note: An Explanation and Elaboration article discusses each checklist item and gives methodological background and published examples of transparent reporting. The STROBE checklist is best used in conjunction with this article (freely available on the Web sites of PLoS Medicine at http://www.plosmedicine.org/, Annals of Internal Medicine at http://www.annals.org/, and Epidemiology at http://www.epidem.com/). Information on the STROBE Initiative is available at www.strobe-statement.org.

For peer review only

BMJ Open

Evaluating the impacts of tiered restrictions introduced in England, during October and December 2020 on COVID-19 cases: A synthetic control study

Journal:	*BMJ Open*
Manuscript ID	bmjopen-2021-054101.R1
Article Type:	Original research
Date Submitted by the Author:	22-Feb-2022
Complete List of Authors:	Zhang, Xingna; University of Liverpool Faculty of Health and Life Sciences, Department of Public Health and Policy<br>Owen, Gwilym; University of Liverpool, Department of Public Health and Policy<br>Green, Mark; University of Liverpool, Geography & Planning<br>Buchan, Iain; University of Liverpool, Department of Public Health, Policy and Systems<br>Barr, Ben ; University of Liverpool, Department of Public Health, Policy and Systems
**Primary Subject Heading**:	Infectious diseases
Secondary Subject Heading:	Health policy, Public health
Keywords:	COVID-19, Health policy < HEALTH SERVICES ADMINISTRATION & MANAGEMENT, PUBLIC HEALTH

SCHOLARONE™
Manuscripts

I, the Submitting Author has the right to grant and does grant on behalf of all authors of the Work (as defined in the below author licence), an exclusive licence and/or a non-exclusive licence for contributions from authors who are: i) UK Crown employees; ii) where BMJ has agreed a CC-BY licence shall apply, and/or iii) in accordance with the terms applicable for US Federal Government officers or employees acting as part of their official duties; on a worldwide, perpetual, irrevocable, royalty-free basis to BMJ Publishing Group Ltd ("BMJ") its licensees and where the relevant Journal is co-owned by BMJ to the co-owners of the Journal, to publish the Work in this journal and any other BMJ products and to exploit all rights, as set out in our licence.

The Submitting Author accepts and understands that any supply made under these terms is made by BMJ to the Submitting Author unless you are acting as an employee on behalf of your employer or a postgraduate student of an affiliated institution which is paying any applicable article publishing charge ("APC") for Open Access articles. Where the Submitting Author wishes to make the Work available on an Open Access basis (and intends to pay the relevant APC), the terms of reuse of such Open Access shall be governed by a Creative Commons licence – details of these licences and which Creative Commons licence will apply to this Work are set out in our licence referred to above.

Other than as permitted in any relevant BMJ Author's Self Archiving Policies, I confirm this Work has not been accepted for publication elsewhere, is not being considered for publication elsewhere and does not duplicate material already published. I confirm all authors consent to publication of this Work and authorise the granting of this licence.

For peer review only

Evaluating the impacts of tiered restrictions introduced in England, during October and December 2020 on COVID-19 cases: A synthetic control study

*Xingna Zhang, Gwilym Owen, Mark Green, Iain Buchan, Ben Barr
Public Health, Policy and Systems, Institute of Population Health, University of Liverpool;
Public Health, Policy and Systems, Institute of Population Health, University of Liverpool;
Department of Geography & Planning, University of Liverpool;
Public Health, Policy and Systems, Institute of Population Health, University of Liverpool;
Public Health, Policy and Systems, Institute of Population Health, University of Liverpool

*xingna.zhang@liverpool.ac.uk
Whelan Building, The Quadrangle,
Liverpool L69 3GB,
United Kingdom

Word count (excluding title page, abstract, references, figures and tables): 3,056.

For peer review only

For peer review only - http://bmjopen.bmj.com/site/about/guidelines.xhtml

ABSTRACT

OBJECTIVES
To analyse the impact on SARS-CoV-2 transmission of Tier 3 restrictions introduced in October and December 2020 in England, compared to Tier 2 restrictions. We further investigate whether these effects varied between small areas by deprivation.

DESIGN
Synthetic control analysis.

SETTING
We identified areas introducing Tier 3 restrictions in October and December, constructed a synthetic control group of places under Tier 2 restrictions, and compared changes in weekly infections over a 4-week period. Using interaction analysis, we estimated whether this effect varied by deprivation and the prevalence of a new variant (B.1.1.7).

INTERVENTIONS
In both October and December, no indoor between-household mixing was permitted in either Tier 2 or 3. In October, no between-household mixing was permitted in private gardens and pubs and restaurants remained open only if they served a 'substantial meal' in Tier 3, whilst in Tier 2 meeting with up to 6 people in private gardens were allowed and all pubs and restaurants remained open. In December, in Tier 3 pubs and restaurants were closed, whilst in Tier 2 only those serving food remained open. The differences in restrictions between Tier 2 and 3 on meeting outside remained the same as in October.

MAIN OUTCOME MEASURE
Weekly reported cases adjusted for changing case-detection rates for neighbourhoods in England.

RESULTS
Introducing Tier 3 restrictions in October and December was associated with a 14% (95% CI 10% to 19%) and 20% (95% CI 13% to 29%) reduction in infections respectively, compared to the rates expected with Tier 2 restrictions only. The effects were similar across levels of deprivation and by the prevalence of the new variant.

CONCLUSIONS
Compared to Tier 2 restrictions, additional restrictions in Tier 3 areas in England had a moderate effect on transmission, which did not appear to increase socioeconomic inequalities in COVID-19 cases.

Article summary

Strengths and limitations of this study

- The synthetic control method for microdata offers a rigorous method for identifying control areas that experienced similar levels of transmission as the intervention areas prior to the introduction of tiered restrictions supporting a casual interpretation of the finding of reduced transmission in areas with greater restrictions following their introduction.
- The use of small area data enabled interaction analysis to estimate whether this effect varied by level of deprivation.
- Our analysis assumes that case detection rates are similar across small areas in each local authority and the relationship between infections and hospitalisations (i.e. the infection hospitalisation rate) remained constant over the study period.

For peer review only - http://bmjopen.bmj.com/site/about/guidelines.xhtml

- If there were differential trends in case detection rates between intervention and control areas this could bias the results.
- There may also be other differences between intervention and control areas, beyond those included in this study, that led to the differences in the trajectory of SARS-CoV-2 infections observed, such as individual or household characteristics

For peer review only

For peer review only - http://bmjopen.bmj.com/site/about/guidelines.xhtml

INTRODUCTION

In Autumn 2020, England experienced a second wave of COVID-19 cases with prevalence increasing 10-fold from 0.1% in August to 1% in October.[1] Reported cases of COVID-19 were unevenly distributed across the country, with areas in the North of England most severely affected. Initially a variety of local restrictions were introduced, which became a standardised three Tier system in October. This was followed by a month-long national lockdown, with tiered restrictions re-introduced in December and a further national lock-down in January 2021.

During the pandemic, evidence has accumulated showing that restrictions such as closing schools, public event bans, and stay-at-home orders, have substantially reduced transmission of SARS-CoV-2.[2] Most evidence is, however, based on national implementation of policies[2,3] and cross-country comparisons,[2] that may not be applicable to restrictions varying across small areas within countries. Much evidence is also based on simulation rather than empirical studies, whereby observed changes in mobility and survey-based indicators of social contact following interventions are used to predict the expected impact of restrictions on cases and hospitalisations using compartmental models.[4] Estimates of intervention impact from such studies may differ from the observed impact on cases, if actual relationships between mobility and transmission following intervention differ from simulation assumptions.

There are also concerns that such restrictions may have differential effects between different socioeconomic contexts. This is because people of disadvantaged communities may not have the same access to outdoor spaces for socialising, there may be differences in the use of restaurants and pubs and differences on the effectiveness of communications aiming to increase compliance with restrictions.[5] Understanding the potential differences in effect of control measures by socioeconomic group is important because the pandemic has disproportionally affected more disadvantaged groups.[6,7] Interventions designed to control the pandemic may further exacerbate these inequalities if we do not evaluate their differential effects and take actions to mitigate any intervention-generated inequalities.[8]

In this study we analyse the impact on SARS-CoV-2 transmission of Tier 3 restrictions introduced in October and December in England, compared to Tier 2 restrictions, where the main differences were additional restrictions on meeting people outdoors and the hospitality sector in Tier 3 areas. We further investigate whether these effects varied between small areas by level of deprivation.

METHODS

Patient and Public Involvement

Patients and public were not involved.

Data and setting

We use UK government data on weekly number of people with at least one positive COVID-19 test result[9] living in 7201 Middle Layer Super Output Areas (MSOAs), between September 2020 and January 2021. MSOAs are standard geographical units used to report statistics in England, with an average population of 8,000. Where there were fewer than 3 cases in any given week the number of cases was supressed. In these situations, we imputed the number of cases, using complete data available at a higher geographical level (Local Authority), so that the sum of cases across MSOAs within a Local Authority (LA) was equal to the total number of weekly cases reported for that LA. In total, 4%

For peer review only - http://bmjopen.bmj.com/site/about/guidelines.xhtml

of the outcome data was imputed (see Appendix 1 for further details). LAs are municipalities covering the whole of England and have largely been the sub-national geographical units used to organise response, testing and control measures during the pandemic.

As trends in reported cases are affected by changes in testing capacity, testing strategy and public behaviour. We adjusted the weekly cases reported for each MSOA by dividing it by a weekly case detection rate estimate in each LA, calculated using a method outlined by Kulu and Dorey (see Appendix 2).[10]

We also measured local area characteristics that could potentially influence transmission and/or effectiveness of control measures. These included the overall score of the English Indices of Multiple Deprivation (IMD) - a composite measure of socioeconomic disadvantage that combines 7 domains of deprivation (income, employment, education, health, crime, barriers to housing & services, and living environment),[11] average number of care home beds per capita from the Care Quality Commission, population density calculated as the mid-2019 population estimates for each MSOA divided by area of the MSOA in hectares from the Office for national statistics,[12,13] the percentage of the over 70 and 7-11 population using 2019's mid-year population estimates from the Office for National Statistics, the proportion of the Black Asian and Minority Ethnic (BAME) groups and the proportion of students from the 2011 Census. To additionally account for potential differences in testing between areas, we used data for LAs on the number of tests per capita in the 4 weeks prior to the intervention available from the UK government COVID-19 dashboard. To account for differences in the prevalence of the new variant B.1.1.7,[14] we included the proportion of positive tests with S-gene failure on PCR testing for each LA from Public Health England.[15]

This time series of MSOA weekly data, area characteristics and linked LA data, was then merged with a dataset indicating the restrictions that each area was under in any given week. Data on restrictions was compiled and made available from the Open Data Institute.[16]

Intervention

In tiered restrictions introduced in both October and December, no mixing between households was permitted indoors in either Tier 2 or Tier 3. The main difference in October was that in Tier 3 people were prohibited from meeting with people outside their household in private gardens and Pubs and restaurants were only allowed to remain open if they were serving a 'substantial meal', whilst in Tier 2 people were allowed to meet with up to 6 people in private gardens and all pubs and restaurants remained open. In December, in Tier 3 pubs and restaurants were closed, whilst in Tier 2 only those serving food remained open. The differences in restrictions between Tier 2 and 3 on meeting outside remained the same as in October. See Appendix 3 for further details.

Analysis

We investigate two intervention time points, weeks commencing 19th October and 7th December 2020. In each period the initial allocation to tiers was announced on Friday and we take the week starting the following Monday as the start of the intervention. We define MSOAs as being in the intervention group if they were in Tier 3 at those time points. We investigate the change in cases in the intervention group, four weeks before and after that time point, compared to a synthetic control group derived from places that entered Tier 2 at the same time. We analyse the two groups based on their initial allocation, even though in October 22% of the MSOAs initially allocated to Tier 2 were later moved to Tier 3. All the MSOAs, apart from those in one local authority (Kent), initially allocated to Tier 3 in December stayed in Tier 3 until the country entered a national lockdown beginning at January 2021. Analysing the groups based on their initial allocation is analogous to an intention to treat analysis in a trial and will provide a more conservative estimate of effect size. This will be less prone

For peer review only - http://bmjopen.bmj.com/site/about/guidelines.xhtml

to bias of selecting places based on their subsequent transition into Tier 3, which in itself would be influenced by the effectiveness of the tiered restrictions.[17]

We apply the synthetic control method for microdata developed by Robbins et al to estimate the intervention effect.[18,19] The synthetic control method is a generalisation of difference-in-difference methods, whereby an untreated version of the treated cases (i.e. a synthetic control) is created using a weighted combination of untreated cases. As the allocation to Tier 3 areas was based on the average level and trend in cases at the LA level this method is able to identify many small areas of Tier 2 that had similar levels and trends in cases as Tier 3 areas before the introduction of restrictions.

To construct the synthetic control group, we derive calibration weights to match the MSOAs in Tier 2 to Tier 3 areas across the four-week period prior to the intervention by local area characteristics described earlier and the corresponding case trends and levels. For the October period we additionally included the number of weeks each area experienced local restrictions before introducing the tiered system. The weighting algorithm derives weights that meet three constraints. Firstly, the sum of weights in the control group equals the number of cases in the intervention group. Secondly, the weighted average of each local area characteristic in the synthetic control group matches those in the intervention group. Lastly, the synthetic control and intervention group also match across all pre-intervention time points in case numbers.[18]

The Average Treatment Effect for the Treated (ATT) is estimated as the difference in cumulative number of cases in the intervention group in the four weeks after the intervention time point, compared to the (weighted) number of cases in the synthetic control group. To estimate the 95% confidence intervals and p-values we apply a permutation procedure, through repeating the analysis through 250 placebo permutations randomly allocating Tier 2 MSOAs to the intervention group.[19] All analysis was performed using R version 4.0.3 and the Microsynth package.[18]

To investigate whether there was a differential effect by socioeconomic group we grouped the MSOAs into 3 equal sized groups (tertiles) by level of deprivation. We then re-ran the weighting algorithm, stratifying the process by IMD tertile. The ATT can be estimated using the calibration weights in a weighted generalised linear model with the binary variable for the intervention group as the exposure. Accounting for the distribution of the outcome data, we fitted a weighted Poisson model with a log link function and the stratified weights, alongside an interaction term between IMD tertile and the intervention indicator.

In December, transmission and potentially the effectiveness of interventions were affected by the emergence of a new variant (B.1.1.7). We used the same approach as above for IMD to investigate differences in the effect of entering Tier 3 in December, based on the prevalence of the new variant in each area.

Sensitivity analysis

To explore sensitivity to different assumptions, we used the confirmed COVID-19 cases instead of wider case-detection rates as our outcome and we found larger effects in both time periods (see Appendix 4). When excluding the Tier 2 MSOA areas located within 20 km of Tier 3 areas we found smaller effects but with high p values (see Appendix 5). This suggests that there may have been some spill-over effects, whereby travel from Tier 3 areas to neighbouring Tier 2 areas contributed to a rise in transmission in neighbouring Tier 2 areas. However, such effects may well have occurred by chance.

RESULTS

For peer review only - http://bmjopen.bmj.com/site/about/guidelines.xhtml

We created a map to show the areas that entered Tier 3 at the two time points (Figure 1). In October the initial Tier 3 areas (391 MSOAs) were entirely in the North West of England. In December, a larger proportion of England (2,858 MSOAs) initially entered Tier 3.

Table 1. The comparison between the Tier 3 and Tier 2 areas at the two intervention time points in the 4 weeks prior to the introduction of the Tiered system.

	October 2020		December 2020	
	**Tier 3**	**Tier 2**	**Tier 3**	**Tier 2**
**Average % estimated case detection rate (confirmed cases / estimated number of infections) in 4 weeks before Tiers introduced**	45	52	41	47
**Average tests per 100,000 per week in 4 weeks before Tiers introduced**	3232	2354	2970	2752
**Weekly infections per 100,000 per week in 4 weeks before Tiers introduced**	723	307	784	355
**Care home beds per 10,000 population**	98	69	86	80
**IMD score**	31	25	26	19
**Population density – people per hectare**	32	51	30	41
**% of population 70+**	14	12	14	14
**% population 7-11 years old**	6	6	6	6
**% BAME**	7	22	12	15
**% *S-gene* target failure (SGTF) in routine *PCR***	NA	NA	23	41
**% students**	3	4	3	3
**Total population**	3,068,261	25,272,230	23,347,218	31,682,197
**Number of MSOAs**	391	2994	2858	3774

We presented summary statistics for areas within each Tier for both time points in Table 1. As would be expected estimated SARS-CoV-2 infection rates were higher in Tier 3 areas prior to the introduction of the tiered system at both points. Tier 3 areas were more deprived on average and had a lower proportion of the population from BAME groups and lower population density. There were no differences in terms of students and care homes. In December the new variant was more prevalent in Tier 2 than Tier 3 areas as measured by the proportion of S-gene target failure (SGTF) in routine PCR. In constructing the synthetic control group, weights were calculated to minimise the difference in each of the variables in Table 1. As an exact match was achieved the weighted average of each of these variables in the control group was identical to the intervention group. A map showing the geographical pattern of these weights is given in Appendix 6.

We mapped out the trend in infection rates in the intervention and synthetic control areas before and after intervention (Figure 2). As an exact match was achieved these trends were identical in the synthetic control and intervention groups in the pre-intervention period. In October the rates were increasing before the tiered system was introduced. They then started to fall, with the drop starting first in the Tier 3 areas. With the second implementation of the tiered system in December infection

For peer review only - http://bmjopen.bmj.com/site/about/guidelines.xhtml

rates were falling whilst the country was in national lockdown, then increased as the national lockdown came to an end and the tiered system was re-introduced. This increase however was slower in the Tier 3 areas compared to the synthetic control. See Appendix 7 for charts displaying the differences between outcomes in the intervention and synthetic control groups compared to 250 placebo permutations.

We compared the estimated effect of Tier 3 restrictions to what would have been expected if Tier 2 restrictions had been applied on those areas (Table 2). In October the introduction of Tier 3 restrictions was estimated to have led to 14% fewer cases (95% CI 10% to 19%), than what would have been the case if Tier 2 restrictions would have been applied. In December, the Tier 3 restrictions are estimated to have led to a slightly greater reduction in cases of 20% (95% CI 13% to 29%).

In the subgroup analysis by deprivation there is a statistically significant effect for each level of deprivation for each time period, suggesting that Tier 3 interventions had an effect across all levels of deprivation. In October the effect estimate tended to be greater the more deprived an area was, although there was a high probability that this difference occurred by chance with high p-values. In December, the effect was similar across all levels of deprivation and no interaction effects were detected again. Including an interaction term between the proportion of cases that were the new variant B.1.1.7 and the intervention group, suggested that the effect of Tier 3 restrictions may have been greater in areas where the new variant was more prevalent (-27%, 95% CIs –35% to –18%), but again the p-values for this interaction were greater than 0.05, indicating substantial uncertainty.

Table 2. Results of the synthetic control analysis – indicating the relative reduction in infections in Tier 3 areas compared to what would have been expected if Tier 2 restrictions had been applied. Interaction analysis shows differences in effect by level of deprivation and prevalence of variant B.1.1.7 indicated by S-gene target failure (SGTF) where quantitative reverse transcriptase PCR is used for COVID-19 diagnosis.

	Percentage change in cases	95% CI		p-value	p-value for interaction in subgroup analysis
		LCL	UCL		
**October 2020** - All Tier 3	-14%	-19%	-10%	<0.001	
Most affluent areas	-10%	-17%	-2%	0.016	
Intermediate deprivation	-15%	-22%	-7%	<0.001	0.354
Most deprived areas	-19%	-29%	-7%	0.003	0.214
**December 2020** - All Tier 3	-20%	-29%	-13%	<0.001	
Most affluent areas	-19%	-24%	-14%	<0.001	
Intermediate deprivation	-26%	-38%	-11%	0.001	0.362
Most deprived areas	-14%	-26%	0%	0.046	0.448
Low SGTF (2%-20%)	-13%	-30%	9%	0.236	
Intermediate SGTF (21%-44%)	-6%	-16%	5%	0.271	0.579
High SGTF (45%-85%)	-27%	-35%	-18%	<0.001	0.174

DISCUSSION

Our study presents timely empirical evidence of the effectiveness of implementing regional tiered restrictions to manage COVID-19 responses. We find more stringent regional restrictions effective at

For peer review only - http://bmjopen.bmj.com/site/about/guidelines.xhtml

reducing infection rates. For both time periods, areas placed in Tier 3 restrictions experienced a moderate reduction in SARS-CoV-2 infections compared to what would have been expected if the same areas had been placed into Tier 2. This is consistent with reporting from the UK Scientific Advisory Group on Emergencies (SAGE) – which concluded in November that the October Tier 3 restrictions had reduced transmission, although they concluded that the effect could not be quantified and that it was unclear if Tier 3 restrictions alone would be sufficient to reduce R below 1.[20] One simulation study predicted that moving into Tier 3 restrictions in England in October reduced the effective reproduction number (R) by 10%[4] which is similar to the effect size we estimate here.

This study adds to the evidence that the additional restrictions on outdoor meeting and the hospitality sector have an important role to play in controlling transmission. The two main differences between Tier 3 and Tier 2 were that people in Tier 3 were not allowed to meet with people outside their household in private gardens and there were greater restrictions on pubs and restaurants. We cannot tell from our analysis whether either additional restrictions, or a combination of both were responsible for the effects we observed. There is, however, some previous evidence indicating that that hospitality settings do play a role in transmission,[21–24] and outdoor proximity is thought to have a low risk of transmission.[25] We find no consistent evidence that the effects differed by levels of deprivation. Whilst broader lockdowns that require people to work from home where possible – might be expected to have differential effects by socioeconomic group, with more disadvantaged groups being less likely to be able to work from home, this is not necessarily the case with restrictions to the hospitality sector. It may be the case that transmission within the hospitality sector occurs at a similar level in more deprived and more affluent neighbourhoods and the restrictions introduced reduce these risks by similar amounts.

The emergence of new more infectious variants of SARS-CoV-2 raises concerns regarding the effectiveness of existing control measures. Increased infectiousness could mean that some activities such as outdoor contact previously seen as low risk could become higher risk. Our results suggest that it is plausible that in the second period of our study, when the new variant B.1.1.7 was more prevalent, the marginal effect of restrictions on outdoor meeting and hospitality settings was relatively greater. There was however, high statistical uncertainty with this finding – possibly inflated by variant surveillance being more accurate in some areas than in others. Further investigation is needed to understand the effectiveness of these restrictions in the presence of new variants.

Our analysis has some limitations. Firstly, we were only able to adjust for variation in the case detection rate using a relatively crude measure estimated at the LA level. This estimate assumes that the infection hospitalisation rate and the infection fatality rate do not vary between places that have similar prevalence of underlying health conditions and do not vary over the study periods. Our analysis also assumes that the case detection rate is constant across MSOAs within each LA. We do however find larger effects when not applying our estimated case detection rate and we also adjusted for differences in the amount of testing carried out in each area. Secondly, although we were able to match areas to ensure a good balance of potential confounding factors prior to the intervention, it is still possible that unmeasured variables could bias the results. Thirdly, we were only able to use data on small neighbourhood areas, rather than on individuals and therefore were unable to investigate how effects of control measures varied by individual or household characteristics.

As countries such as the UK continue the battle to control COVID-19 cases, with large regional differences in transmission, tiered restrictions as well as national lockdowns will likely be needed to reduce infection levels whilst sufficient effective vaccination coverage is achieved. At present the UK government's plans for exiting the current lock down are for a staged easing of restrictions at the same time across the whole of England.[26] Concerns have been raised about this – one size fits all – approach, given that high infection rates persist – particularly in more deprived areas.[27] Our analysis

For peer review only - http://bmjopen.bmj.com/site/about/guidelines.xhtml

indicates that tiered restrictions in outdoor gathering and in the hospitality sector are effective at moderately reducing the growth of cases and could be part of an effective strategy for reducing geographical differences in transmission risk as we emerge from the pandemic.

For peer review only

For peer review only - http://bmjopen.bmj.com/site/about/guidelines.xhtml

Author contribution statement

B.B. conceived the original Idea, devised the project, the main conceptual ideas and proof outline. X.Z. and B.B. carried out the data analysis. X.Z. drafted the manuscript and designed the figures with support from G.O. and B.B.. G.O. aided in improving the model specification, and M.G. aided in interpreting the results and commented on the manuscript. I.B. aided in supervising the project and commented on the manuscript. All authors provided critical feedback and helped to shape the research manuscript.

Conflicts of interest statement

We have no conflicts of interest to disclose.

Ethics statements

Patient consent for publication

Not required.

Ethics approval

University Research Ethics Committee review is not required by the University of Liverpool for the secondary analysis of data which have been anonymised by an external party and are provided to the research team in a fully anonymised format.

Data sharing statement

All the data we used are publicly accessible and statistical code is available from the authors upon request.

Acknowledgement

This study is funded by the National Institute for Health Research (NIHR) Health Protection Research Unit in Gastrointestinal Infections, a partnership between Public Health England, the University of Liverpool and the University of Warwick. Grant No: NIHR ref NIHR200910. The views expressed are those of the author(s) and not necessarily those of the NIHR, Public Health England or the Department of Health and Social Care. BB is also supported by the NIHR Applied Research Collaboration North West Coast (ARC NWC). GO is supported by the NIHR School for Public Health Research. IB is supported by NIHR Senior Investigator award. The views expressed in this publication are those of the author(s) and not necessarily those of the NIHR or the Department of Health and Social Care.

For peer review only

For peer review only - http://bmjopen.bmj.com/site/about/guidelines.xhtml

References

1 Office for National Statistics. Coronavirus (COVID-19) Infection Survey, UK: 30 October 2020. 2020.https://www.ons.gov.uk/peoplepopulationandcommunity/healthandsocialcare/conditionsanddiseases/bulletins/coronaviruscovid19infectionsurveypilot/30october2020

2 Li Y, Campbell H, Kulkarni D, *et al.* The temporal association of introducing and lifting non-pharmaceutical interventions with the time-varying reproduction number (R) of SARS-CoV-2: a modelling study across 131 countries. *Lancet Infect Dis* 2021;**21**:193–202. doi:10/ghgjmv

3 Davies NG, Kucharski AJ, Eggo RM, *et al.* Effects of non-pharmaceutical interventions on COVID-19 cases, deaths, and demand for hospital services in the UK: a modelling study. *Lancet Public Health* 2020;**5**:e375–85. doi:10/gg2376

4 Davies NG, Barnard RC, Jarvis CI, *et al.* Association of tiered restrictions and a second lockdown with COVID-19 deaths and hospital admissions in England: a modelling study. *Lancet Infect Dis* 2020;**0**. doi:10/ghrbvd

5 Davidson EM, Liu JJ, Bhopal R, *et al.* Behavior Change Interventions to Improve the Health of Racial and Ethnic Minority Populations: A Tool Kit of Adaptation Approaches. *Milbank Q* 2013;**91**:811–51. doi:10.1111/1468-0009.12034

6 Daras K, Alexiou A, Rose TC, *et al.* How does vulnerability to COVID-19 vary between communities in England? Developing a Small Area Vulnerability Index (SAVI). *J Epidemiol Community Health* Published Online First: 4 February 2021. doi:10.1136/jech-2020-215227

7 McKinley JM, Cutting D, Anderson N, *et al.* Association between community-based self-reported COVID-19 symptoms and social deprivation explored using symptom tracker apps: a repeated cross-sectional study in Northern Ireland. *BMJ Open* 2021;**11**:e048333.

8 Lorenc T, Petticrew M, Welch V, *et al.* What types of interventions generate inequalities? Evidence from systematic reviews. *J Epidemiol Community Health* 2013;**67**:190–3. doi:10.1136/jech-2012-201257

9 Official UK Coronavirus Dashboard. https://coronavirus.data.gov.uk/details/cases (accessed 28 Jan 2021).

10 Kulu H, Dorey P. Infection rates from Covid-19 in Great Britain by geographical units: A model-based estimation from mortality data. *Health Place* 2021;**67**:102460. doi:10.1016/j.healthplace.2020.102460

11 Ministry of Housing C& LG. *English indices of deprivation 2019: research report*. 2019. https://www.gov.uk/government/publications/english-indices-of-deprivation-2019-research-report

12 All data related to Population estimates by output areas, electoral, health and other geographies, England and Wales: mid-2019 - Office for National Statistics. https://www.ons.gov.uk/peoplepopulationandcommunity/populationandmigration/populationestimates/bulletins/annualsmallareapopulationestimates/mid2019/relateddata (accessed 22 Feb 2022).

13 Middle Layer Super Output Areas (December 2001) Boundaries EW BFC. https://geoportal.statistics.gov.uk/datasets/middle-layer-super-output-areas-december-2001-boundaries-ew-bfc/explore?location=52.950000,-2.000000,6.65 (accessed 22 Feb 2022).

For peer review only - http://bmjopen.bmj.com/site/about/guidelines.xhtml

14 Prime Minister's Office, 10 Downing Street. Prime Minister announces Tier 4: 'Stay At Home' Alert Level in response to new COVID variant. 2020.https://www.gov.uk/government/news/prime-minister-announces-tier-4-stay-at-home-alert-level-in-response-to-new-covid-variant (accessed 25 Jan 2021).

15 Kirby T. New variant of SARS-CoV-2 in UK causes surge of COVID-19. *Lancet Respir Med* 2021;**0**. doi:10/ghwffp

16 Tennison J. Local coronavirus restrictions in England. Google Docs. https://docs.google.com/spreadsheets/d/1HBVmvsQXrkQgySW_OiTQdrS8WGCXqgWnmZ43PPi0XgY/edit?usp=embed_facebook (accessed 28 Jan 2021).

17 Cole SR, Platt RW, Schisterman EF, *et al.* Illustrating bias due to conditioning on a collider. *Int J Epidemiol* 2010;**39**:417–20. doi:10.1093/ije/dyp334

18 Robbins MW, Davenport S. microsynth: Synthetic Control Methods for Disaggregated and Micro-Level Data in R. *J Stat Softw Vol 1 Issue 2 2021* Published Online First: 14 January 2021.https://www.jstatsoft.org/v097/i02

19 Robbins MW, Saunders J, Kilmer B. A Framework for Synthetic Control Methods With High-Dimensional, Micro-Level Data: Evaluating a Neighborhood-Specific Crime Intervention. *J Am Stat Assoc* 2017;**112**:109–26. doi:10/f9746k

20 SAGE 67 minutes: Coronavirus (COVID-19) response, 12 November 2020. GOV.UK. https://www.gov.uk/government/publications/sage-67-minutes-coronavirus-covid-19-response-12-november-2020 (accessed 2 Feb 2021).

21 Thompson H, Mousa A, Dighe A, *et al.* Report 38: SARS-CoV-2 setting-specific transmission rates: a systematic review and meta-analysis. 2020. doi:10.25561/84270

22 Koh WC, Naing L, Chaw L, *et al.* What do we know about SARS-CoV-2 transmission? A systematic review and meta-analysis of the secondary attack rate and associated risk factors. *PLOS ONE* 2020;**15**:e0240205. doi:10/ghzjhf

23 Fisher KA. Community and Close Contact Exposures Associated with COVID-19 Among Symptomatic Adults ≥18 Years in 11 Outpatient Health Care Facilities — United States, July 2020. *MMWR Morb Mortal Wkly Rep* 2020;**69**. doi:10/ghbtpr

24 Qiu X, Nergiz AI, Maraolo AE, *et al.* Defining the role of asymptomatic and pre-symptomatic SARS-CoV-2 transmission - a living systematic review. *Clin Microbiol Infect Off Publ Eur Soc Clin Microbiol Infect Dis* Published Online First: 20 January 2021. doi:10/ghzjhd

25 Bulfone TC, Malekinejad M, Rutherford GW, *et al.* Outdoor Transmission of SARS-CoV-2 and Other Respiratory Viruses: A Systematic Review. *J Infect Dis* doi:10/ghp8pd

26 COVID-19 Response - Spring 2021. GOV.UK. https://www.gov.uk/government/publications/covid-19-response-spring-2021 (accessed 1 Mar 2021).

27 Is England's exit plan from lockdown a road to nowhere? The BMJ. 2021.https://blogs.bmj.com/bmj/2021/02/25/is-englands-exit-plan-from-lockdown-a-road-to-nowhere/ (accessed 1 Mar 2021).

For peer review only - http://bmjopen.bmj.com/site/about/guidelines.xhtml

For peer review only

For peer review only - http://bmjopen.bmj.com/site/about/guidelines.xhtml

Figure 1. Location of areas that entered Tier 3 (yellow) and Tier 2 (purple) at the two intervention time points.

Figure 2. The trend in case rates with their 95% confidence intervals in the Tier 3 areas and in the synthetic control group.

For peer review only

For peer review only - http://bmjopen.bmj.com/site/about/guidelines.xhtml

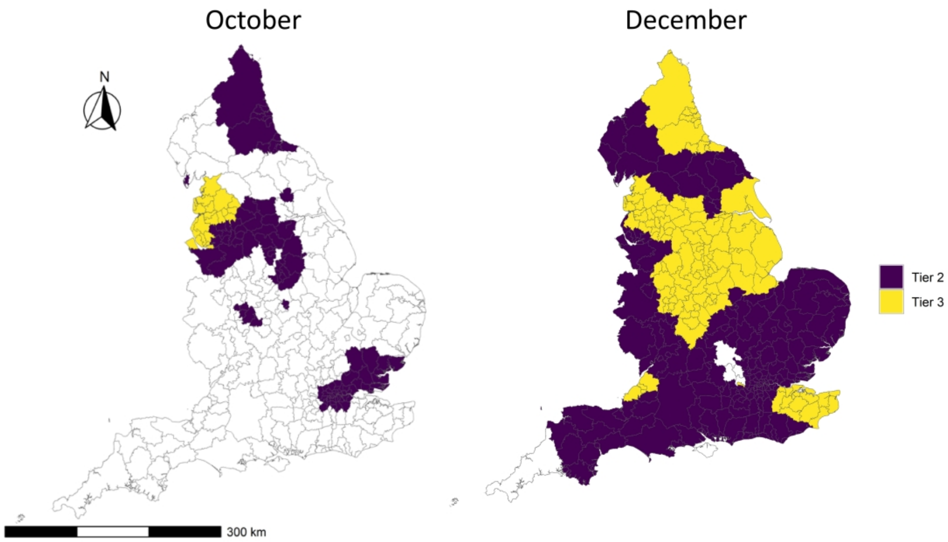

Figure 1. Location of areas that entered Tier 3 (yellow) and Tier 2 (purple) at the two intervention time points.

338x190mm (300 x 300 DPI)

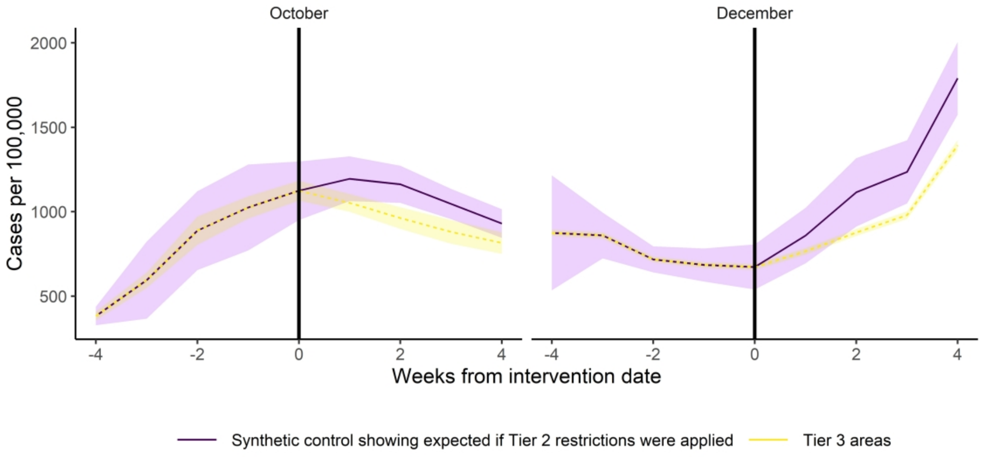

Figure 2. The trend in case rates with their 95% confidence intervals in the Tier 3 areas and in the synthetic control group.

338x190mm (300 x 300 DPI)

Appendix 1. Comparison of case numbers at both MSOA and LA levels before and after imputation.

Table A. Summary statistics of the number of cases at both MSOA and LA levels before and after imputation.

		Minimum	1st Quartile	Median	Mean	3rd Quartile	Maximum	Standard deviation	Missing	Number of observations
October										
MSOA	Before	3	9	17	21.73	28	1063	24.31	2141	28324
	After	0	8	16	20.29	27	1063	24.02	0	30465
LA	Before	2	269	508	868.5	1049	4291	920.60	0	1089
	After	2	269	508	868.5	1049	4291	920.60	0	1089
December										
MSOA	Before	3	10	18	26.68	33	340	25.31	1679	58009
	After	0	10	18	25.97	32	340	25.30	0	59688
LA	Before	15	244.8	507	800.9	946.2	8566	910.36	0	59688
	After	15	244.8	507	800.9	946.2	8566	910.36	0	59688

Appendix 2. Calculating Local Authority level case detection rates.

We use a method proposed by Kulu and Dorey to calculate local authority specific infection rates from data on observed and expected deaths and hospitalisations from Covid-19.[10] The true infection rate was estimated from Covid-19 for each local authority as the number of observed deaths divided by the sum of the expected deaths for each age and sex sub-group, if everyone was infected. The expected deaths for each local authority was calculated by multiplying the population estimates for each age and sex group by the infection fatality rates for that sub-group. The same process was applied using hospitalisations instead of deaths. We use the Infection Fatality and Infection Hospitalisation Rates estimated by Knock et al.[28]

In order to account for varying rates of morbidity between local authorities, following Kulu and Dorey[10] we apply a multiplier of chronic disease prevalence to the expected deaths and hospitalisation for each local authority. The idea being that in areas with higher rates of chronic disease, there would be expected to be higher COVID-19 Infection Fatality and Infection Hospitalisation Rates. Chronic disease was measured as the proportion of the population that had at least one admission to hospital with a diagnosis of cardiovascular disease, chronic respiratory disease, diabetes or chronic kidney disease recorded in their hospital record, which we found in previous research to be highly predictive of COVID-19 mortality.[6] To calculate this multiplier we estimated the increased risk of COVID-19 mortality and hospitalisation associated with chronic illness for each local authority compared to the average using Poisson regression models.

The method outlined above was applied to 7 day moving averages of deaths and hospitalisation for each local authority. As hospital admissions and deaths occur sometime after initial infection, in order to estimate the true infection rate at a specific point in time we need to know the lag between initial case identification and both hospitalisation and death. To estimate this, we ran a set of regression models with observed hospital admissions or deaths as the dependent variable and with observed cases with a range of lags as the predictor variable. We tested lags of 1 to 20 days for hospitalisations and 7 to 30 days for deaths with each different lag tested in a separate model. We determined the best fit lag by selecting the model with the lowest Akaike Information Criterion (AIC). This was 8 days for hospitalisations and 15 days for deaths.

We then divided the 7 day's rolling average of observed cases for each day by the estimated number infected calculated separately using hospitalisation data and deaths data and then took the average

For peer review only - http://bmjopen.bmj.com/site/about/guidelines.xhtml

of the output of the two methods as our measure of case detection rate. Where there was missing data for either hospitalisations or deaths we took the estimated number infected using the method where there was complete data.

Appendix 3. Differences in Tiered restrictions.

Tiered restrictions were introduced at two points in time. Firstly in October LAs in England, were placed in one of three Tiers with restrictions of increasing stringency.26 Tier 1 had the fewest restrictions, groups of up to 6 people were allowed to meet indoors or outdoors. In Tier 2, people were prohibited from mixing inside with individuals outside of their households, were only allowed to meet with up to 6 people outdoors, and pubs and restaurants had to close between 10pm and 5am. In Tier 3, people were additionally prohibited from meeting with people outside their household in private gardens. Pubs and restaurants were only allowed to remain open if they were acting as restaurants and serving a 'substantial meal'. The Tier to which an area was allocated to was based on the average rate of change in case numbers and pressure on the health service across the LA.27 Although explicit criteria have not been published and there is some evidence of pressure from local politicians influencing decisions.28 Following this initial tiered system the whole of England was moved into a month-long national lock down from the 5th November before a return to a three-tier system at the beginning of December.29 This new tiered system had some differences in the restrictions for residents in each tier. The main difference was that in this second period in Tier 2 areas pubs and restaurants were only allowed to stay open if they were serving 'substantial meals', whilst in Tier 3 areas pubs and restaurants had to close except for providing takeaway food. The areas in each tier remained the same until 19th of December, when some areas were re-assigned to a new 'Tier 4' on that day because of rapidly rising case numbers in certain areas, thought to largely be due to a new more infectious variant of the virus, B.1.1.7.11 In Tier 4 areas the guidance was to stay at home except for essential journeys. In both periods and in both Tier 2 and Tier 3, no mixing between households was permitted indoors.

Appendix 4. Replicating analysis only using confirmed cases as an outcome.

We found larger effects when using confirmed COVID-19 cases instead of wider case-detection rates as our outcome in both time periods.

Table B. The comparison of the number of confirmed cases between the Tier 3 and synthetic control Tier 2 areas during the two intervention periods, with lower (LCL) and upper (UCL) 95% confidence limits.

	Percentage change in cases	LCL	UCL	p-value
**October** - All Tier 3	-21%	-28%	-16%	<0.001
**December** - All Tier 3	-28%	-31%	-25%	<0.001

Appendix 5. Sensitivity tests of the spatial spill-over effect.

A concern with localised restrictions in the control of COVID-19 is that there may be spill-over effects, whereby, people move from areas of high restrictions to take advantage of lower restrictions (e.g. visiting restaurants and pubs) in neighbouring areas, increasing transmission in those areas and reducing the overall effectiveness of a system of differential local restrictions. In order to test the spatial spill-over effect between the treatment (Tier 3) and comparison (Tier 2) group, we exclude the Tier 2 MSOA areas locating within 20 km of the treatment group. The distance between MSOA areas is measured by the Euclidean distance of the population weighted centroids of MSOA areas (Data source is ONS Geography Open Data https://geoportal.statistics.gov.uk/datasets/b0a6d8a3dc5d4718b3fd62c548d60f81_0).

For peer review only - http://bmjopen.bmj.com/site/about/guidelines.xhtml

When excluding the Tier 2 MSOA areas located within 20 km of Tier 3 areas we found smaller effects but with high p values (see Table B). This suggests that there may have been some spill-over effects, whereby travel from Tier 3 areas to neighbouring Tier 2 areas contributed to a rise in transmission in neighbouring Tier 2 areas. However, such effects may well have occurred by chance.

Table C. The comparison of the number of cases between the Tier 3 and synthetic control Tier 2 areas during the two intervention periods, excluding the Tier 2 MSOA areas locating within 20 km of the treatment group.

	Percentage change in cases	LCL	UCL	p-value
**October -** All Tier 3	-12%	-25%	3%	0.112
**December -** All Tier 3	-12%	-28%	-2%	0.128

Figure A. Location of areas that entered Tier 3 (yellow) and Tier 2 (purple) at the two intervention time points, excluding the Tier 2 MSOA areas locating within 20 km of the treatment group.

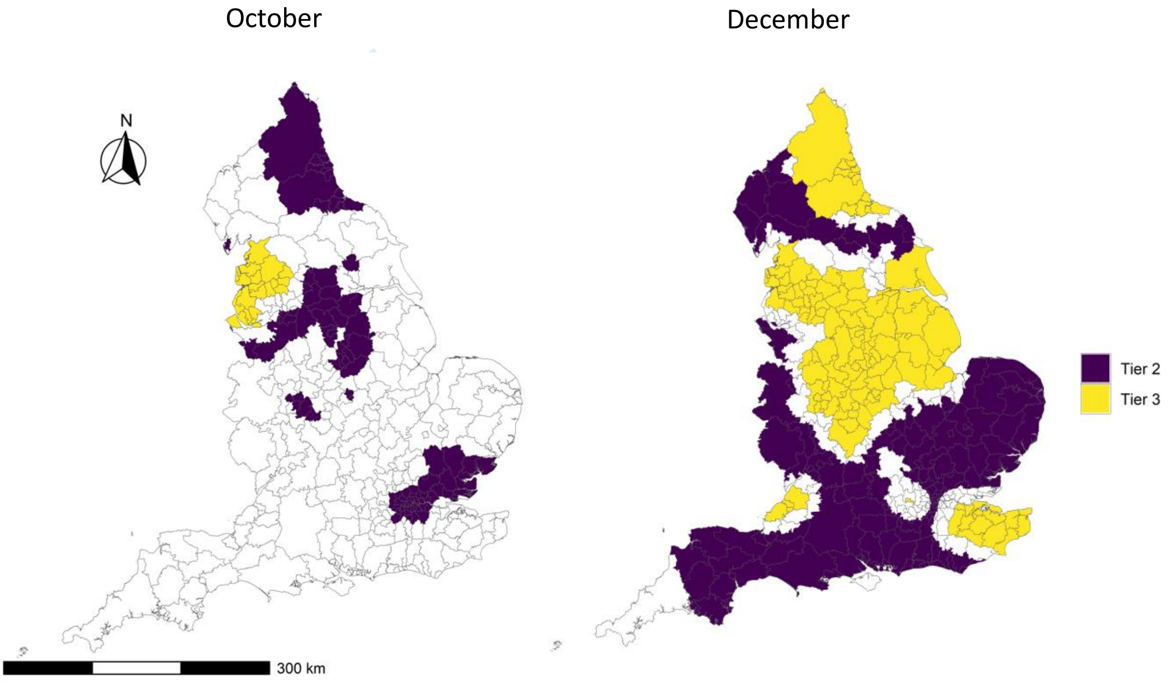

Appendix 6. Weighting of Tier 2 areas used to construct synthetic control group.

Figure B. Weighting of areas that entered Tier 3 (red) and Tier 2 (other colours) to construct synthetic control group at the two intervention time points.

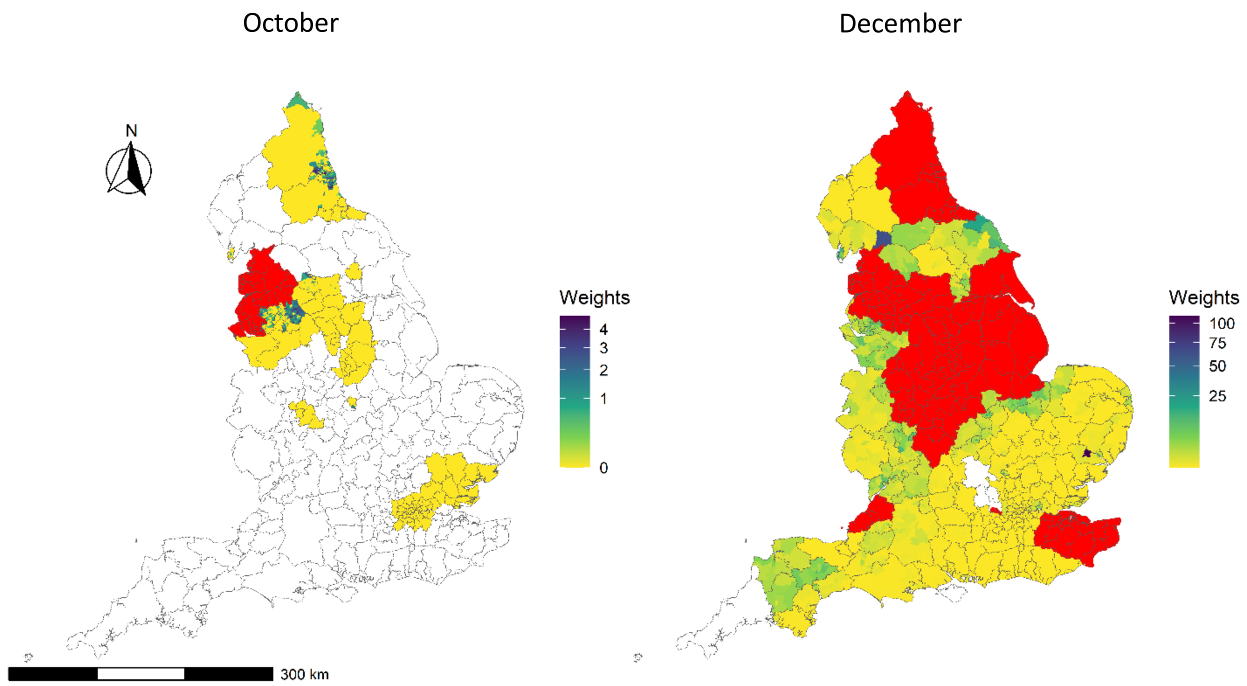

Appendix 7. Placebo permutations.

Comparison of Tier 3 cases following introduction of tiered restrictions in October compared to a synthetic control.

Figure C. October all areas.

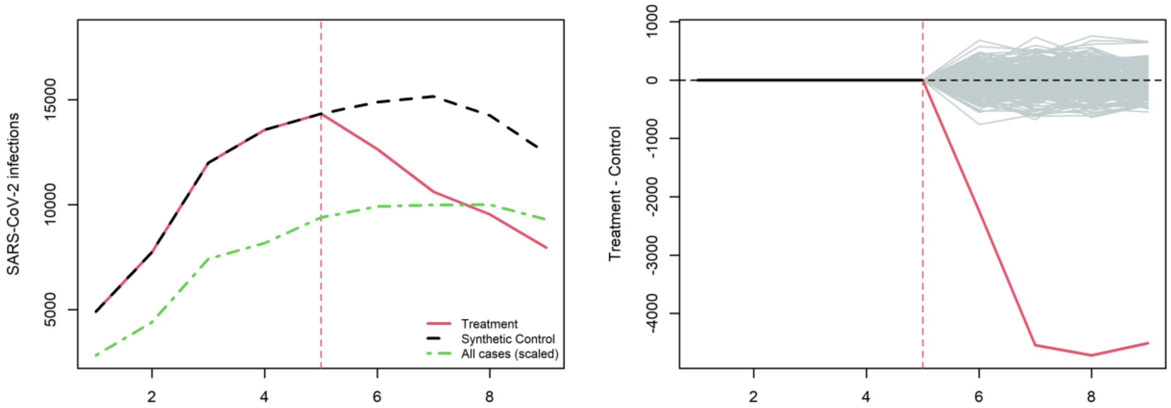

Comparison of Tier 3 cases following introduction of tiered restrictions December compared to a synthetic control.

Figure D. December all areas.

For peer review only - http://bmjopen.bmj.com/site/about/guidelines.xhtml

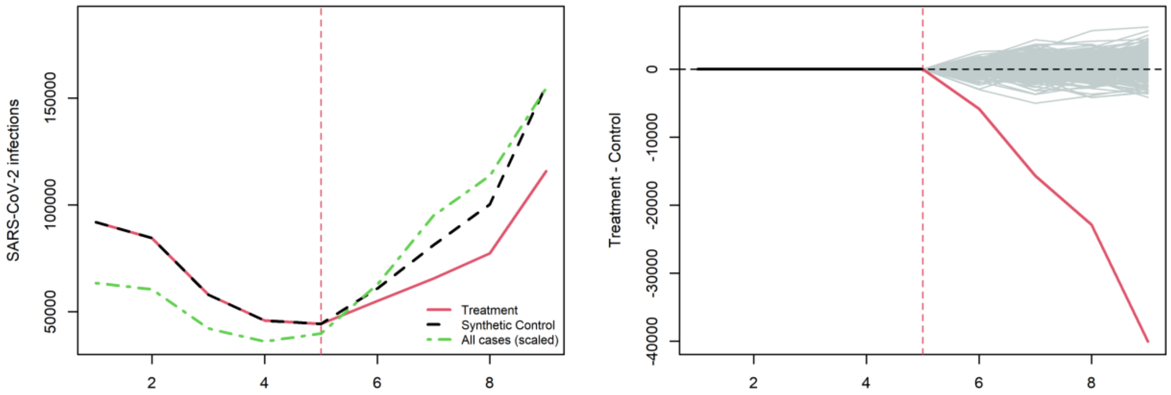

For peer review only - http://bmjopen.bmj.com/site/about/guidelines.xhtml

STROBE 2007 (v4) Statement—Checklist of items that should be included in reports of *cross-sectional studies*

**Section/Topic**	**Item #**	**Recommendation**	**Reported on page #**
**Title and abstract**	1	(*a*) Indicate the study's design with a commonly used term in the title or the abstract	1
		(*b*) Provide in the abstract an informative and balanced summary of what was done and what was found	2-3
**Introduction**			
Background/rationale	2	Explain the scientific background and rationale for the investigation being reported	4
Objectives	3	State specific objectives, including any prespecified hypotheses	4
**Methods**			
Study design	4	Present key elements of study design early in the paper	4
Setting	5	Describe the setting, locations, and relevant dates, including periods of recruitment, exposure, follow-up, and data collection	4-5
Participants	6	(*a*) Give the eligibility criteria, and the sources and methods of selection of participants	4-6
Variables	7	Clearly define all outcomes, exposures, predictors, potential confounders, and effect modifiers. Give diagnostic criteria, if applicable	5-6
Data sources/ measurement	8*	For each variable of interest, give sources of data and details of methods of assessment (measurement). Describe comparability of assessment methods if there is more than one group	4-5, 7
Bias	9	Describe any efforts to address potential sources of bias	*6*
Study size	10	Explain how the study size was arrived at	7
Quantitative variables	11	Explain how quantitative variables were handled in the analyses. If applicable, describe which groupings were chosen and why	4-8
Statistical methods	12	(*a*) Describe all statistical methods, including those used to control for confounding	5-8
		(*b*) Describe any methods used to examine subgroups and interactions	8
		(*c*) Explain how missing data were addressed	4
		(*d*) If applicable, describe analytical methods taking account of sampling strategy	
		(*e*) Describe any sensitivity analyses	6
**Results**			

For peer review only

Participants	13*	(a) Report numbers of individuals at each stage of study—eg numbers potentially eligible, examined for eligibility, confirmed eligible, included in the study, completing follow-up, and analysed	7
		(b) Give reasons for non-participation at each stage	7
		(c) Consider use of a flow diagram	
Descriptive data	14*	(a) Give characteristics of study participants (eg demographic, clinical, social) and information on exposures and potential confounders	7
		(b) Indicate number of participants with missing data for each variable of interest	
Outcome data	15*	Report numbers of outcome events or summary measures	7-8
Main results	16	(*a*) Give unadjusted estimates and, if applicable, confounder-adjusted estimates and their precision (eg, 95% confidence interval). Make clear which confounders were adjusted for and why they were included	8
		(*b*) Report category boundaries when continuous variables were categorized	8
		(*c*) If relevant, consider translating estimates of relative risk into absolute risk for a meaningful time period	
Other analyses	17	Report other analyses done—eg analyses of subgroups and interactions, and sensitivity analyses	8
**Discussion**			
Key results	18	Summarise key results with reference to study objectives	8-9
Limitations	19	Discuss limitations of the study, taking into account sources of potential bias or imprecision. Discuss both direction and magnitude of any potential bias	9
Interpretation	20	Give a cautious overall interpretation of results considering objectives, limitations, multiplicity of analyses, results from similar studies, and other relevant evidence	8-9
Generalisability	21	Discuss the generalisability (external validity) of the study results	9
**Other information**			
Funding	22	Give the source of funding and the role of the funders for the present study and, if applicable, for the original study on which the present article is based	10

*Give information separately for cases and controls in case-control studies and, if applicable, for exposed and unexposed groups in cohort and cross-sectional studies.

Note: An Explanation and Elaboration article discusses each checklist item and gives methodological background and published examples of transparent reporting. The STROBE checklist is best used in conjunction with this article (freely available on the Web sites of PLoS Medicine at http://www.plosmedicine.org/, Annals of Internal Medicine at http://www.annals.org/, and Epidemiology at http://www.epidem.com/). Information on the STROBE Initiative is available at www.strobe-statement.org.

For peer review only
